# Automated detection and quantification of bone disorganization in hypophosphatasia before and after asfotase alfa: A case report

**DOI:** 10.1016/j.bonr.2026.101963

**Published:** 2026-10-23

**Authors:** Simon Zhang, Cat Shore-Lorenti, Cherie Chiang, Frances Milat, Peter R. Ebeling, Roger Zebaze

**Affiliations:** a Monash University School of Clinical Sciences at Monash Health, Clayton, Victoria, Australia; b Austin Health, Heidelberg, Victoria, Australia; c Monash Health, Clayton, Victoria, Australia; d Hudson Institute of Medical Research, Clayton, Victoria, Australia; e Division of Endocrinology, Department of Medicine, Temerty Faculty of Medicine, University of Toronto, Toronto, Canada; f Li Ka Shing Knowledge Institute, St. Michael's Hospital, Unity Health Toronto, Toronto, Ontario, Canada

**Keywords:** Hypophosphatasia, Bone disorganization, Asfotase alfa, Alignogram, Imaging biomarker

## Abstract

Conventional imaging poorly captures bone disorganization, a pathological feature of hypophosphatasia (HPP). This single-patient case report applied ALIGNOGRAM to previously published open-access distal radius radiographs from a 16-year-old with severe HPP before and after six months of asfotase alfa. It detected overt, subtle, and covert lesions and quantified reduced lesion burden post-treatment. Residual disorganization persisted. These findings support preliminary technical feasibility, with independent validation in larger prospective cohorts required to establish diagnostic and treatment-response validity.

## 1 Introduction

According to the prevailing view, healthy bone is characterised by adequate mineralized matrix reflected by normal bone mineral density (BMD), as well as preserved structural integrity, including normal cortical thickness and density and a well-connected trabecular architecture (Kanis, 2002; Zebaze et al., 2010). Although BMD and bone microarchitecture are important determinants of skeletal strength, they do not fully explain fracture susceptibility in many skeletal disorders (Zebaze and Ebeling, 2023; Zebaze et al., 2023). An additional determinant is how the components that make up bone are organized relative to one another. Bone quantity reflects how much bone material is present, structure describes its architecture, and organization describes how its components are arranged and integrated. Normal BMD or apparently preserved architecture does not necessarily produce strong bone if those components are poorly aligned or abnormally arranged. Bone disorganization refers to this loss of normal spatial organization (Zebaze and Ebeling, 2023; Zebaze et al., 2023).

Operationally, ALIGNOGRAM assesses this property on conventional radiographs by quantifying abnormalities in the spatial relationships and X-ray attenuation of neighbouring bone pixels (Zebaze et al., 2023). Bone disorganization may arise when disease processes disrupt the formation, arrangement, maintenance or remodeling of bone tissue, potentially impairing load transfer, creating local stress concentrations and promoting microdamage, thereby contributing to skeletal fragility independently of bone quantity (Zebaze and Ebeling, 2023; Zebaze et al., 2023; Hernandez and van der Meulen, 2017).

Hypophosphatasia (HPP) provides a particularly relevant example because defective mineralization can disturb not only the amount of mineralized bone but also the organization of the tissue that is formed. Pathogenic variants in the *ALPL* gene reduce tissue-nonspecific alkaline phosphatase activity, resulting in accumulation of inorganic pyrophosphate (PPi), a potent inhibitor of hydroxyapatite mineralization (Hasegawa et al., 2017). Elevated PPi impairs mineral deposition within osteoid and disrupts crystal nucleation along collagen fibrils, resulting in heterogeneous and abnormally organized bone tissue (Villa-Suárez et al., 2021). Thus, skeletal fragility in HPP cannot necessarily be understood simply from how much bone or mineral is present; how the components of the bone matrix are organized relative to one another may also be important. This distinction is particularly relevant because BMD in HPP can vary considerably and does not by itself capture the underlying mineralization defect or tissue organization.

Conventional imaging techniques such as dual-energy X-ray absorptiometry (DXA) primarily measure BMD, while high-resolution peripheral quantitative computed tomography (HR-pQCT) characterizes bone microarchitecture (Zebaze et al., 2023; Choksi et al., 2018). Although these modalities provide valuable structural information, they do not directly assess bone tissue disorganization, which may contribute to fracture burden independently of bone mass. Clinical scoring systems developed for disorders such as HPP, including the Radiographic Global Impression of Change (RGIC) and the Rickets Severity Score (RSS), provide semi-quantitative assessment of radiographic abnormalities but remain dependent on subjective visual interpretation, are susceptible to inter-observer variability, and are not readily integrated into automated image analysis workflows (Whyte et al., 2018; Thacher et al., 2019; Gillies et al., 2016). Furthermore, reliance on visual assessment limits the detection of subtle or covert abnormalities that may precede overt radiographic changes (Wang and Guo, 2021).

To address these limitations, we previously developed ALIGNOGRAM, an automated image-analysis platform that quantifies bone disorganization from conventional radiographs (Zebaze et al., 2023). In patients with atypical femoral fractures (AFFs), ALIGNOGRAM identified overt disorganized lesions and distinguished affected individuals from healthy controls. Importantly, it also detected subtle abnormalities within contralateral femora that appeared radiographically normal, distinguishing these femora from those of healthy controls (Zebaze et al., 2023). These findings are supported by the observation that up to 62.5% of AFFs are bilateral (Probyn et al., 2015), suggesting that structural abnormalities may exist before becoming visually apparent.

In the present study, we investigated whether automated disorganization analysis could be applied to HPP, a disorder in which abnormal tissue organization is a fundamental pathological feature. Using plain distal radius radiographs obtained before and after six months of asfotase alfa therapy in a 16-year-old patient with severe HPP (Bowden and Adler, 2018), we evaluated the ability of ALIGNOGRAM to detect overt, subtle, and covert disorganized lesions and to quantify treatment-associated longitudinal changes in bone disorganization.

## 2 Case

### 2.1 Patient/images

Disorganization analysis was performed using ALIGNOGRAM 1.0 (Zebaze et al., 2023). Wrist radiographs from a 16-year-old male with severe childhood-onset HPP were analysed before and after six months of treatment with asfotase alfa (2 mg/kg administered subcutaneously three times weekly) (Bowden and Adler, 2018; Bowden and Foster, 2018). The radiographs were obtained from an open-access dataset available under a Creative Commons Attribution-NonCommercial (CC BY-NC) license (Bowden and Adler, 2018). Permission to reproduce the selected images was obtained from Dove Medical Press Ltd., and the study protocol was approved by the Monash Health Human Research Ethics Committee.

### 2.2 Quantification of disorganization

Disorganization analysis was restricted to the bone matrix, excluding the articular and periosteal surfaces. For longitudinal anatomical matching, the distal radial endplate (distal articular surface) was used as the reference landmark. Anatomically corresponding regions of the distal radial metaphysis were analysed before treatment and after six months of therapy by selecting the region at the same distance from the distal radial endplate on both radiographs. Images were oriented using the distal articular surface as the anatomical reference, with this surface positioned perpendicular to the longitudinal direction of analysis. Magnification was used after quantitative analysis for visualization, characterization and illustration of detected lesions and did not alter the quantitative measurements.

The analysis used previously acquired radiographs for which complete original acquisition metadata were unavailable; therefore, identical projection and acquisition parameters between baseline and six months could not be independently verified. Anatomically corresponding regions were matched using the distal radial endplate and local osseous landmarks. Analysis used the original radiographic images, and exposure was not manually adjusted for quantitative measurement. Anatomical matching, standardized orientation and greyscale normalization may not eliminate all effects of differences in radiographic acquisition parameters.

Following selection of the region of interest, all subsequent ALIGNOGRAM image processing and quantitative disorganization analysis were performed automatically, without further manual intervention.

Bone disorganization was quantified using OAS1.0 software (Melbourne, Australia), as previously described (Zebaze et al., 2023). To minimize the potential influence of differences in image quality on this longitudinal analysis, original grayscale pixel intensities on a 0–255 scale were converted to a relative percentage scale from 0 to 100 using min–max normalization within each co-registered region of interest:

$$A_{\mathrm{Ni}}=100*\left(\frac{A_{i}-{\mathrm{Min}}_{A}}{{\mathrm{Max}}_{A}-{\mathrm{Min}}_{A}}\right)$$

where A_Ni_ represents the normalized attenuation value of pixel i, A_i_ its original attenuation value, and Max_A_ and Min_A_ the maximum and minimum attenuation values, respectively, within the co-registered region of interest. This preprocessing step standardizes the grayscale range across images before quantitative pixel-level analysis.

The software then performs pixel-by-pixel analysis, quantifying local disorganization according to two key features: (i) spatial mislocation of a pixel relative to its neighbors and (ii) differences in normalized X-ray attenuation (greyscale intensity). These features are combined to generate a disorganization value (DV) for every pixel. The analysis is conducted on neighbouring pixels within a moving symmetric analysis window centered on each pixel of interest. The first layer includes the eight immediate neighbors in a 3 × 3 grid, while the second layer encompasses an additional sixteen next-nearest neighbors within a 5 × 5 window, totaling 24 surrounding pixels. By considering this extended neighborhood, the method captures more complex local patterns and enhances the sensitivity of disorganization detection. The analysis window moves across the image, analyzing successive pixels until the entire selected bone region has been covered.

To identify disorganized pixels (i.e., local outliers), a threshold was applied to the matrix of 24 neighbouring pixels using the formula.

(1) DV>Q3+1.5×IQR,

where Q3 represents the third quartile and IQR the interquartile range of neighbouring DV values (Barbato et al., 2011). Pixels exceeding this critical disorganization value (CDV) were flagged as disorganized. The threshold was adopted unchanged from the previously published and validated ALIGNOGRAM methodology, which demonstrated that CDV tracked experimentally imposed disorganization and discriminated between clinical groups (Zebaze et al., 2023). It was not optimized for this case, and alternative thresholds were not tested; therefore, the sensitivity of the present findings to threshold choice remains unknown. This operational threshold identifies local outliers and should not be interpreted as an established biological or clinical cutoff in HPP. Connectivity analysis was then used to group adjacent disorganized pixels into distinct regions of abnormality, denoted as disorganized lesions.

ALIGNOGRAM generated three complementary outputs: (i) color-coded lesion maps superimposed on the original radiograph to facilitate visual localization; (ii) pixel-wise disorganization profiles along the length of the analysed bone demonstrated on an ALIGNOGRAM curve that plots pixel position along the bone (x-axis) against their respective DV (y-axis); and (iii) quantitative metrics describing regional and overall disorganization, including density (%) of disorganized pixels and areas.

### 2.3 Compositional analysis

Following lesion detection, the system characterizes the pixel composition underlying each disorganized lesion. To ensure statistically reliable comparisons, the size of each disorganized region is first compared with that of the adjacent non-disorganized reference region. When the reference region is more than four times larger than the disorganized region (ratio > 4:1), the lesion is classified as a small disorganized area. Such regions are considered insufficient for robust statistical comparison because of the increased risk of biased estimates and inflated Type I and Type II errors (Linden and Samuels, 2013). Consequently, compositional analysis is not performed.

When the size ratio is ≤4:1, the lesion is classified as a large disorganized area, and compositional analysis proceeds. Attenuation histograms are generated for both the disorganized region and the adjacent non-disorganized bone to compare their attenuation distributions. Non-overlapping histogram distributions indicate that disorganization is associated with either abnormally lucent (hypodense) or abnormally opaque (hyperdense) pixels. Quantile-based comparisons (e.g., the 5th and 95th percentiles) are then used to identify the specific abnormal pixels responsible for the observed differences.

### 2.4 Statistical analysis

Results are presented as absolute values, standard deviations (SDs), medians, interquartile ranges (IQR), and percentage changes. Percent mean changes from baseline were calculated using the formula:

(2) $$\text{Percent change}=\left(\text{Post}-\text{treatment}-\mathrm{Pre}-\text{treatment}\right)/\mathrm{Pre}-\text{treatment}\times 100.$$

As this was a single-patient case study, no statistical comparisons were performed.

### 2.5 Visual assessment and lesion classification

Radiographs were first assessed visually by three expert readers (RZ, CSL and SZ) without knowledge of the subsequent ALIGNOGRAM findings. ALIGNOGRAM analysis was then performed independently, and the visual and quantitative findings were subsequently compared. Lesions identified by both expert visual assessment and ALIGNOGRAM and readily visible radiographically were classified as overt. Lesions identified by both but considered difficult to appreciate visually were classified as subtle. Lesions not identified during the initial expert visual assessment performed without ALIGNOGRAM, but subsequently detected and localized by ALIGNOGRAM, were classified as covert. Classification required consensus among three reviewers (RZ, CSL and SZ), and the final classifications were reviewed and approved by all authors.

All lesions automatically detected by ALIGNOGRAM were color-coded and localized on the dual-display screen, and the corresponding regions on the original radiographs were visually reviewed and confirmed by three authors (RZ, CSL and SZ). The findings were subsequently reviewed and agreed upon by all authors. This lesion-confirmation review was performed with knowledge of the ALIGNOGRAM localization and was distinct from the initial visual assessment. Inter-reader agreement was not formally quantified, and this case report was not designed as a diagnostic-accuracy reader study.

### 2.6 Automated detection of disorganized lesions

ALIGNOGRAM automatically performed pixel-level analysis of the distal radius radiographs from a 16-year-old male with HPP, identifying and quantifying disorganization across the bone matrix.

At baseline, 644 of 40,515 pixels (1.59%) exceeded the critical disorganization threshold (DV > Q3 + 1.5 × IQR) and were classified as disorganized. These disorganized pixels formed 204 distinct clusters, corresponding to a disorganized pixel density (DPD) of 1.59% and 5.04 disorganized areas per 1000 pixels.

Each automatically detected disorganized area was displayed using the ALIGNOGRAM split-screen interface, enabling immediate visual verification of the corresponding lesion location (Fig. 1, Fig. 2, Fig. 3). Based on visual assessment by the authors, the detected lesions were classified into three categories: overt lesions when readily visible during initial assessment (Fig. 1); subtle lesions when identified but difficult to appreciate visually (Fig. 2); and covert lesions when not identified during initial assessment but subsequently detected and localized by ALIGNOGRAM (Fig. 3).

**Fig. 1:**
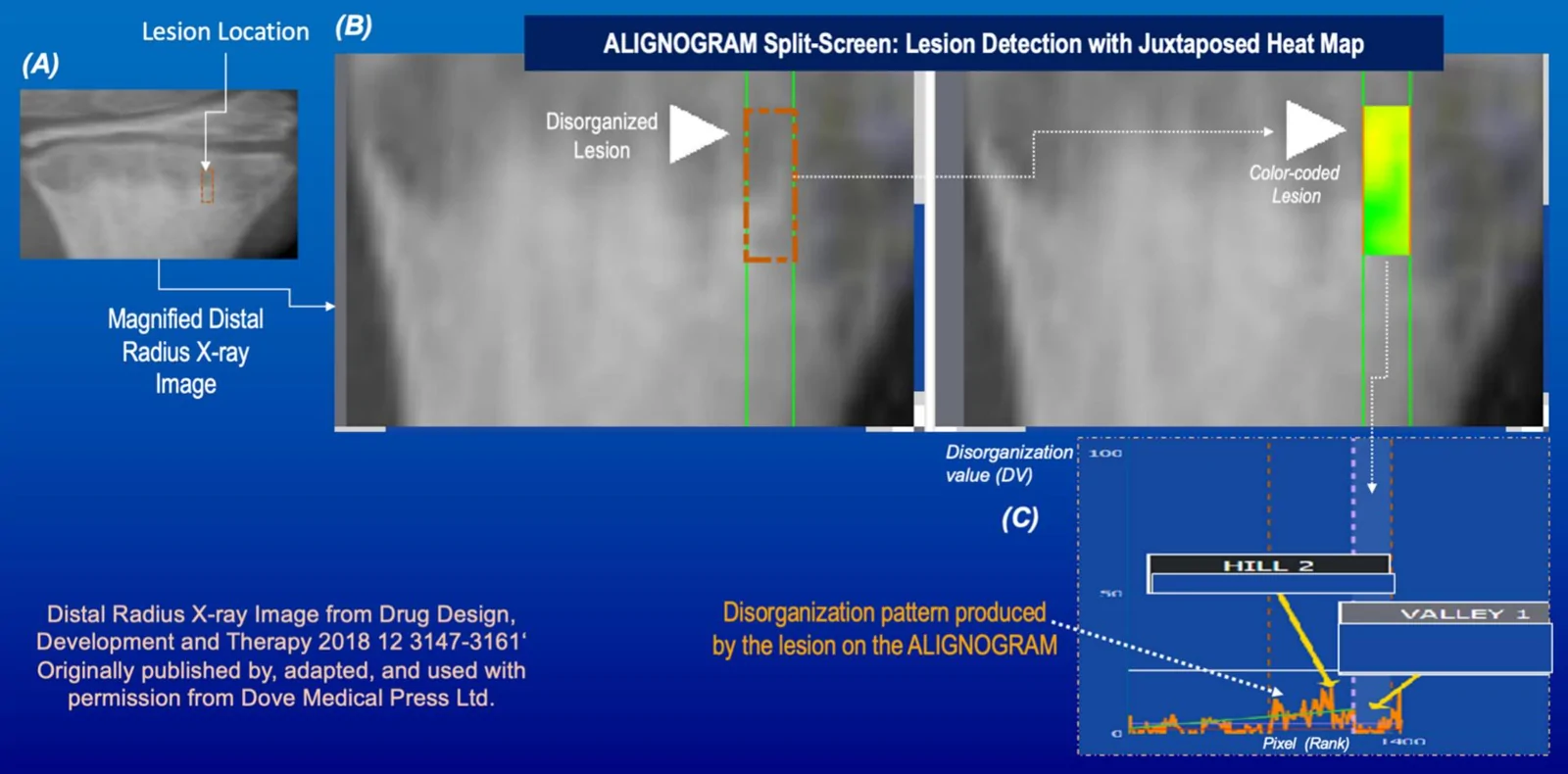
Automated detection of an overt disorganized lesion with the corresponding quantitative disorganization profile. (A) Original distal radius radiograph showing the region of interest. The automatically detected lesion is outlined by the orange dashed rectangle. (B) ALIGNOGRAM split-screen display of the detected lesion. The left panel shows the lesion on the original radiograph, while the right panel presents the corresponding color-coded disorganization map. White arrows indicate the lesion location. (C) Corresponding ALIGNOGRAM disorganization profile. The lesion is associated with a focal increase in disorganization, appearing as a peak followed by a trough in the profile. Vertical dashed lines indicate the automatically identified lesion boundaries.

**Fig. 2:**
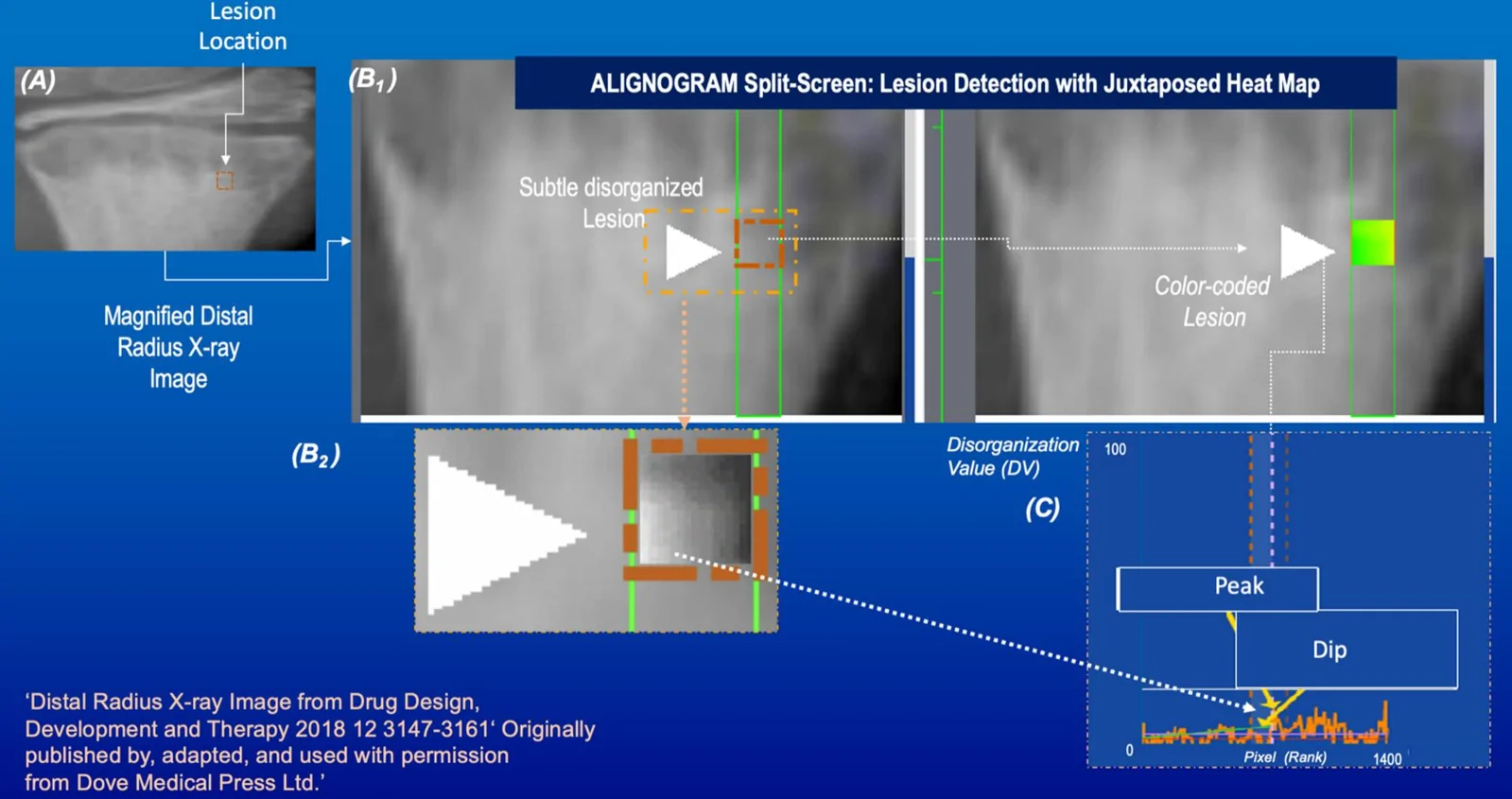
Automated detection of a subtle disorganized lesion with the corresponding quantitative disorganization profile. (A) Original distal radius radiograph showing the region of interest. The automatically detected lesion is outlined by the orange dashed rectangle. The lesion is inconspicuous on routine visual inspection. (B) ALIGNOGRAM split-screen display of the detected lesion. (B1) Original radiograph showing the automatically localized lesion. The right panel presents the corresponding color-coded disorganization map. White arrows indicate the lesion location. (B2) Magnified and contrast-enhanced view of the same region, demonstrating the subtle structural abnormality. (C) Corresponding ALIGNOGRAM disorganization profile. The lesion is associated with a focal increase in disorganization, producing a gradual peak within the profile. Vertical dashed lines indicate the automatically identified lesion boundaries.

**Fig. 3:**
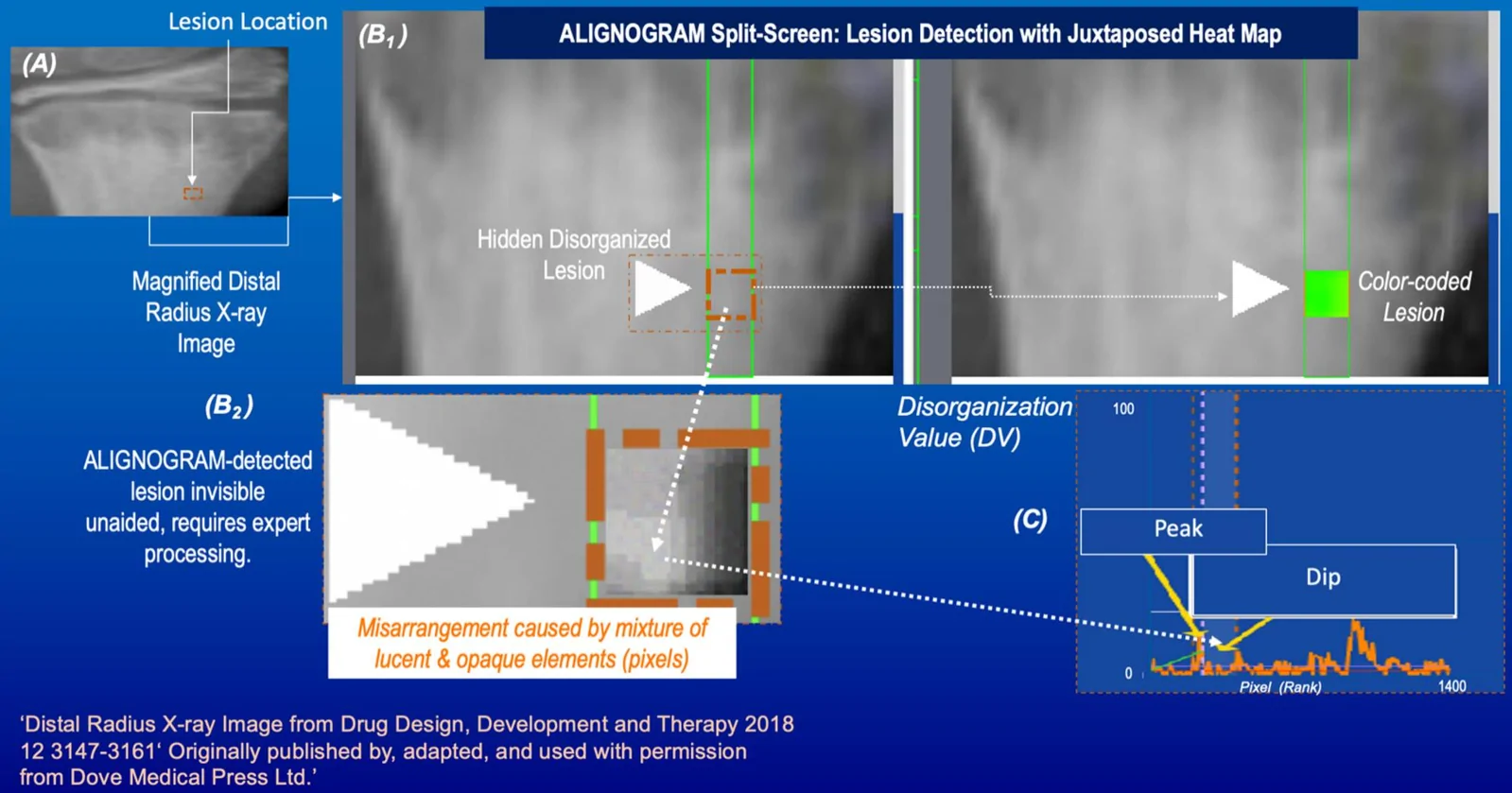
Automated detection of a covert disorganized lesion with the corresponding quantitative disorganization profile. (A) Original distal radius radiograph showing the region of interest. The automatically detected lesion is outlined by the orange dashed rectangle. The abnormality was not identified on initial visual assessment. (B) ALIGNOGRAM split-screen display of the detected lesion. The left panel shows the automatically localized lesion on the original radiograph together with a magnified, contrast-enhanced view of the same region, in which the structural abnormality becomes apparent. The right panel presents the corresponding color-coded disorganization map. White arrows indicate the lesion location. (C) Corresponding ALIGNOGRAM disorganization profile. The lesion is associated with a focal increase in disorganization, characterised by a peak followed by a trough in the profile. Vertical dashed lines indicate the automatically identified lesion boundaries.

For each detected lesion, ALIGNOGRAM generated a color-coded lesion map and a corresponding quantitative disorganization profile, providing complementary visual and quantitative characterization of the abnormality.

The representative lesions shown in Fig. 1, Fig. 2, Fig. 3 illustrate that focal changes in the quantitative disorganization profile consistently corresponded to lesion location across the spectrum of radiographic conspicuity. Additional representative examples are provided in the Supplementary material.

### 2.7 Assessment of treatment-associated changes in disorganization

Following six months of asfotase alfa therapy, quantitative disorganization analysis demonstrated a marked reduction in overall bone disorganization, as quantified by ALIGNOGRAM. The number of disorganized pixels decreased by 80%, from 644 to 129, corresponding to a fivefold reduction in the DPD from 1.59% to 0.32%. Similarly, the number of discrete disorganized lesions decreased by 47.5%, from 204 to 107, reducing lesion density from 5.04 to 2.64 areas per 1000 image pixels. The proportion of large disorganized areas also declined from 52.94% at baseline to 29.91% after treatment, representing a relative reduction of 43.5%.

### 2.8 Reduction in severity of highly disorganized lesions

The four most severely disorganized lesions demonstrated substantial improvement following six months of therapy (Table 1). The lesion sizes decreased by an average of 58.1%, accompanied by reductions in the median, maximum, and standard deviation of disorganization values of 40%, 37%, and 43%, respectively.

**Table 1:** Temporal changes in disorganization severity features of the four most misarranged areas between baseline and six-month therapy in a 16-year-old adolescent with hypophosphatasia.

Characteristics of the most disorganized areas	Baseline Median (max—min)	After six months of therapy Median (max—min)	Percentage reduction in disorganization severity
**Mean** disorganization value (arbitrary unit)	10.2 (10.2–9.78)	6.61 (7.39–6.26)	−39.85%
**Maximum** disorganization value (arbitrary unit)	27.11 (27.57–26.89)	18.56 (20.28–18.44)	−37.44%
**Average standard deviation** of disorganization value (arbitrary unit)	6.87 (6.97–6.85)	4.46 (5.93–4.42)	−42.61%
**Area** (pixels number)	2137 (2150–2125)	1175 (1175–850)	−58.11%

These quantitative improvements are illustrated in Fig. 4, Fig. 5, which compare the four most severely affected lesions before and after treatment respectively.

**Fig. 4:**
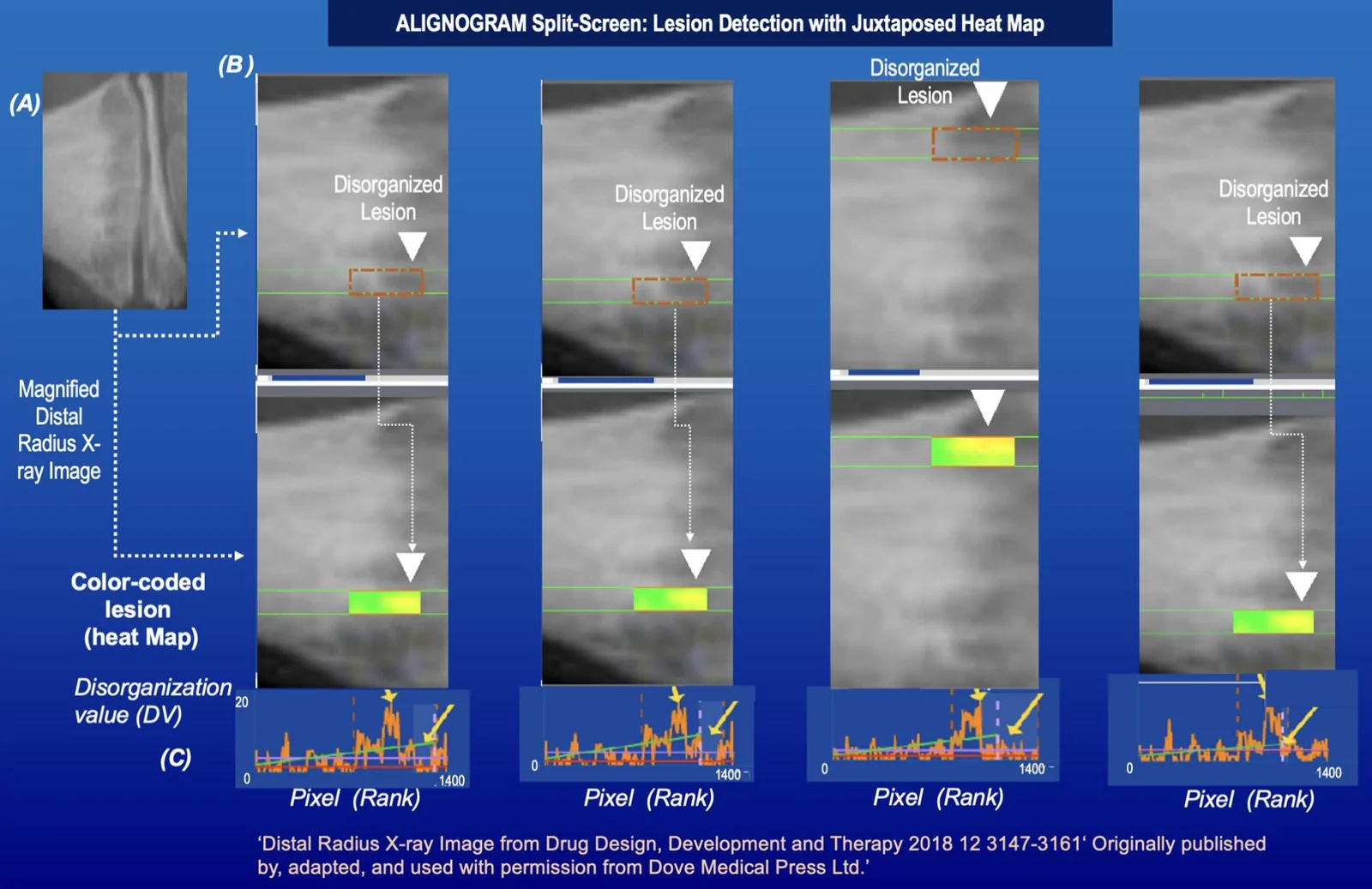
Four highly disorganized lesions identified at baseline with corresponding quantitative disorganization profiles. (A) Original distal radius radiograph. (B) ALIGNOGRAM split-screen display of the four most severely disorganized lesions. The upper panels show the automatically localized lesions on the original radiograph, while the lower panels present the corresponding color-coded disorganization maps. White arrows indicate the lesion locations. (C) Corresponding ALIGNOGRAM disorganization profiles for each lesion. The x-axis represents pixel position along the analysed bone, and the y-axis represents the disorganization value (DV). Vertical dashed lines indicate the automatically identified lesion boundaries.

**Fig. 5:**
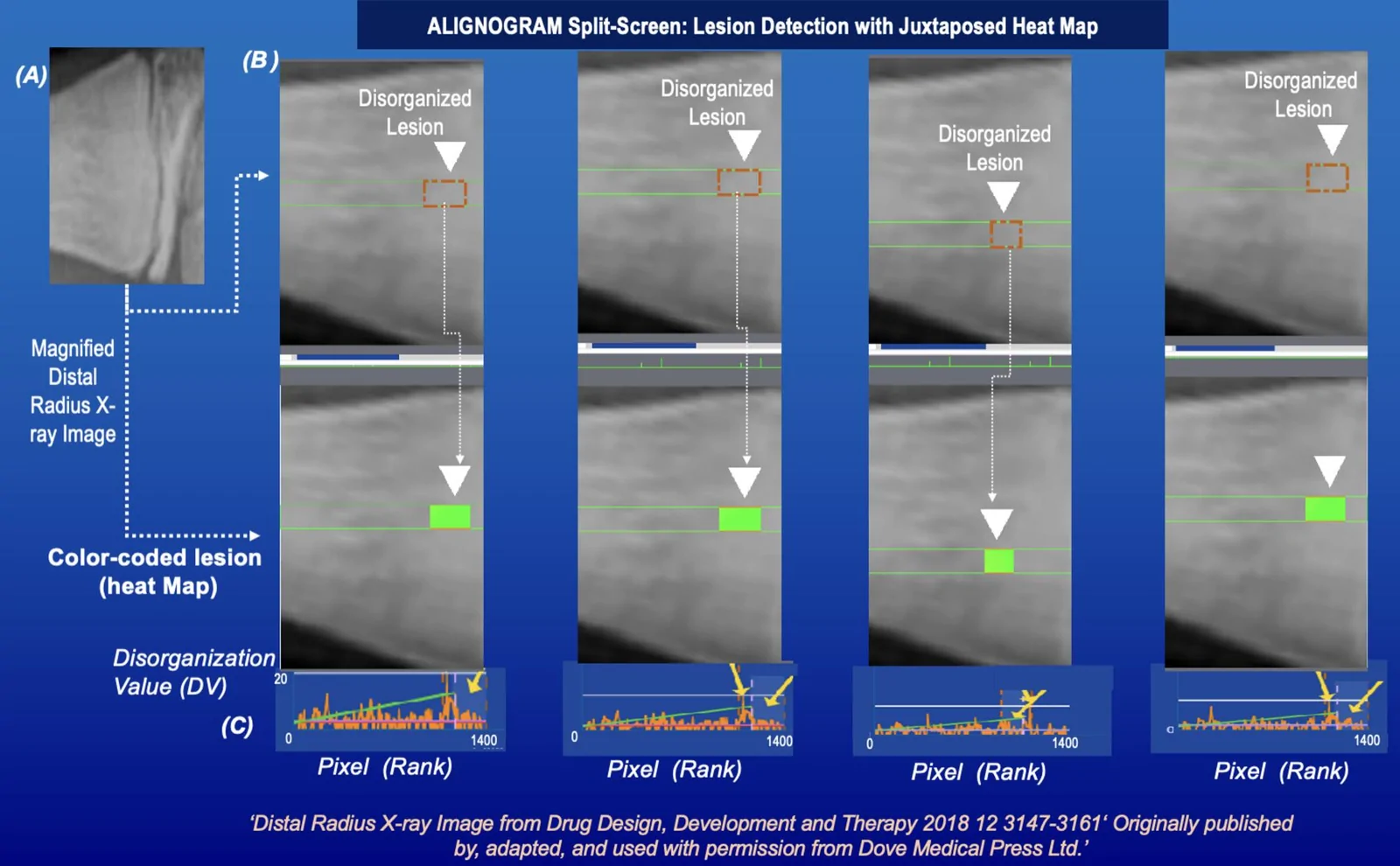
Four highly disorganized lesions after six months of asfotase alfa therapy with corresponding quantitative disorganization profiles. (A) Original distal radius radiograph obtained after six months of therapy. (B) ALIGNOGRAM split-screen display showing corresponding lesions on the original radiograph (upper panels) and color-coded disorganization maps (lower panels). White arrows indicate lesion locations. (C) Corresponding ALIGNOGRAM disorganization profiles.

The ALIGNOGRAM profile provides a quantitative representation of the local disorganization associated with each lesion. Vertical dashed lines indicate the automatically identified lesion boundaries. Greater lesion severity is characterised by larger deviations and increased irregularity within the disorganization profile, whereas flatter and more homogeneous profiles indicate lower levels of structural disorganization. Compared with baseline (Fig. 4), the disorganization profiles are flatter and less irregular, consistent with reduced structural disorganization following six months of therapy.

### 2.9 Compositional analysis: treatment-associated changes in lesion composition

Therapy was associated with marked changes in the composition of disorganized lesions (Fig. 6). At baseline, 59.3% of lesions assessed for compositional analysis contained abnormal lucent (hypodense) and/or opaque (hyperdense) pixel populations. Following six months of asfotase alfa therapy, these abnormal compositional features were no longer detected. Residual disorganization consisted of altered spatial arrangement of otherwise normally attenuating pixels, while abnormal lucent and opaque pixel populations were no longer detected in the regions assessed.

**Fig. 6:**
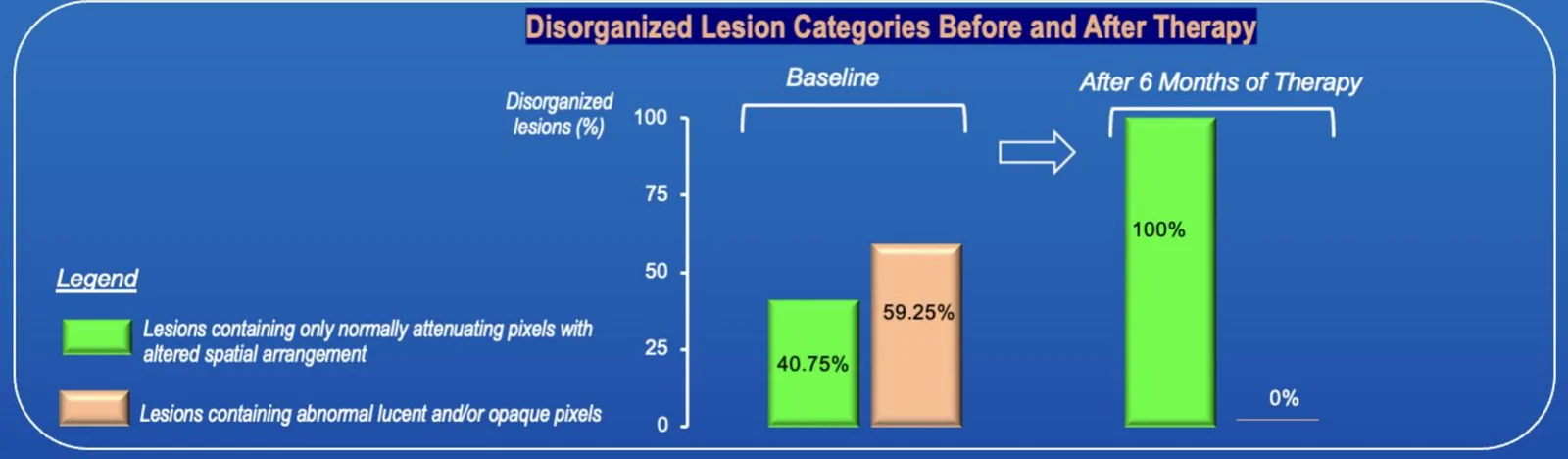
Compositional analysis of disorganized lesions before and after six months of asfotase alfa therapy. Percentages represent the proportion of lesions calculated at each time point using the total number of lesions eligible for compositional analysis as the denominator. (A) At baseline, 59.25% of assessed lesions contained abnormal lucent (hypodense) and/or opaque (hyperdense) pixel populations, whereas 40.75% contained only normally attenuating pixels with altered spatial arrangement. (B) After six months of therapy, no assessed lesions contained abnormal lucent or opaque pixel populations; all assessed lesions (100%) contained only normally attenuating pixels with altered spatial arrangement.

## 3 Discussion

This single-patient case report demonstrates the feasibility of automated detection and quantification of disorganized bone lesions in a patient with HPP from conventional radiographs. In addition to identifying lesions, ALIGNOGRAM quantified treatment-associated changes in lesion burden and radiographic attenuation patterns during six months of asfotase alfa therapy. The abnormally lucent and opaque components detected at baseline were no longer detected after treatment, while residual disorganization attributable to altered pixel arrangement persisted. The observed reductions in lesion burden and severity support further investigation of quantitative disorganization analysis for assessing treatment-associated longitudinal changes. Although the present report describes a single longitudinal HPP case, it forms part of a broader evidence base that includes prior quantitative validation of the methodology and a separate published longitudinal HPP study demonstrating treatment-associated changes preceding corresponding changes on conventional radiography or bone scintigraphy, with further supportive evidence reported across HPP and other skeletal disorders (Zebaze et al., 2023, Zebaze et al., 2025; Witanachchi et al., 2026; Maheshwari et al., 2026). Nevertheless, larger prospective studies are required to establish HPP-specific diagnostic performance, treatment-response validity and clinical utility.

ALIGNOGRAM is intended to complement expert radiographic interpretation by automatically identifying regions of interest for targeted review. This attention-guidance approach may improve lesion detection by directing both expert and non-expert readers to specific areas warranting closer inspection. In this case, three authors (RZ, CSL and SZ) reviewed the corresponding regions after ALIGNOGRAM localization, and the findings were subsequently reviewed and agreed upon by all authors. All flagged regions were considered abnormal during targeted review. Because the confirmation review used the same radiographs and knowledge of the ALIGNOGRAM findings, it provides anatomical correlation but does not establish independent diagnostic validity or comparative performance against expert or non-expert readers.

Previous work demonstrated that ALIGNOGRAM can detect both overt disorganized lesions, such as atypical femoral fractures, and covert abnormalities within contralateral non-fractured femora (Zebaze et al., 2023). The present study extends these observations to HPP, demonstrating automated detection of lesions across a continuum of radiographic visibility. Lesions were classified as overt, subtle, or covert according to their visibility on the original radiograph and the degree of image enhancement required for visual recognition. It is important to emphasize that this framework is descriptive rather than a validated diagnostic classification. It illustrates differences in lesion conspicuity, while the biological and clinical significance of detected regions requires independent validation.

The identification of covert lesions has several potential clinical implications. Radiographs interpreted as normal may still contain previously unrecognized abnormalities, contributing to false-negative assessments and underestimation of disease burden. Their implications for diagnosis, disease progression and longitudinal assessment require further investigation. Earlier recognition of structural abnormalities may facilitate more timely diagnosis, which is particularly relevant in HPP, where diagnostic delays of 4–10 years have been reported (Villa-Suárez et al., 2021). More broadly, these findings raise important questions regarding the clinical significance of radiographic abnormalities that are imperceptible on conventional visual assessment but detectable using advanced image analysis and artificial intelligence-assisted methods. Such approaches may enhance the diagnostic capability of conventional radiography by revealing structural abnormalities beyond the limits of unaided human interpretation.

Beyond lesion detection, quantitative disorganization analysis has the potential to monitor disease behavior and treatment response. Six months of asfotase alfa therapy was associated with marked reductions in both the proportion of disorganized pixels and the aggregation of these pixels into larger lesions, while disorganization profiles became flatter and more homogeneous. Pixel analysis identified abnormally lucent (lower-attenuation) and abnormally opaque (higher-attenuation) components, which largely resolved following treatment. These radiographic attenuation patterns may reflect heterogeneity in mineralization, and their underlying tissue composition cannot be determined from radiographic attenuation alone. Although clinically meaningful thresholds for disorganization remain to be established, longitudinal changes in quantitative disorganization metrics may prove useful for assessing treatment efficacy in HPP.

Bone density and structural assessments were not included in this study, as the primary objective was to evaluate bone disorganization. Furthermore, conventional measures such as BMD and trabecular bone score (TBS) have limited utility in hyperostotic disorders and HPP (Kanis, 2002; Zebaze and Ebeling, 2023; Choksi et al., 2018). However, this case did not directly compare ALIGNOGRAM with BMD, TBS or advanced imaging, and cannot establish comparative performance or the biological nature of individual lesions.

While this study focused solely on HPP, bone disorganization represents a pathogenic feature shared across several skeletal disorders (Zebaze and Ebeling, 2023; Zebaze et al., 2023). Accordingly, automated disorganization analysis may have broader applications in conditions such as osteogenesis imperfecta, osteopetrosis, neurofibromatosis, chronic kidney disease–mineral and bone disorder, diabetes-related bone disease, and X-linked hypophosphataemia, where objective assessment of skeletal organization remains a potential area for future investigation.

This study has several limitations. First, it represents a single-patient longitudinal case report at one anatomical site and two time points. Thus, these findings by themselves cannot establish the diagnostic performance, treatment response validity or clinical utility of ALIGNOGRAM. Second, the present case does not have an independent reference standard, such as complementary imaging or histology, or corresponding biochemical and functional outcomes, to confirm the biological and clinical significance of individual ALIGNOGRAM-detected regions. Expert assessment of the same radiographs provides anatomical correlation but is not an independent biological validation. This limitation applies to individual findings in the present patient rather than to the analytical performance of the underlying methodology, which has previously undergone controlled pixel-level experimental validation, precision testing and in-vivo clinical evaluation. Future prospective studies incorporating complementary imaging and clinical outcomes, and where feasible histological correlation, are warranted. Third, although the reduction in disorganization occurred during six months of asfotase alfa therapy, causality cannot be established from this single case. Growth, skeletal maturation, image positioning, exposure differences and natural temporal variability cannot be completely excluded as contributors. Although anatomical matching, standardized image orientation and greyscale normalization were applied, their ability to control these influences was not quantified, and residual confounding could not be excluded. Moreover, although quantitative measurements were generated using a predefined algorithm and previously published methodology, independent external replication and blinded reader validation will be important to further validate these findings.

This study demonstrates the feasibility of combining standard radiographs with automated image analysis to detect and quantify bone disorganization in HPP. The findings reveal a spectrum of lesion visibility, ranging from overt to covert abnormalities, including abnormalities that may not be readily appreciable by visual assessment alone. ALIGNOGRAM quantified reductions in disorganized pixels, lesion burden and lesion severity after six months of asfotase alfa therapy, supporting the feasibility of assessing treatment-associated longitudinal changes. This is particularly relevant in HPP, where bone-specific quantitative imaging tools for assessing skeletal abnormalities and their longitudinal changes are limited, while standard radiographs are widely available and routinely used in clinical care. Larger prospective studies incorporating independent imaging and clinical outcomes are required to validate these findings and establish diagnostic and treatment-response performance.

## CRediT authorship contribution statement

**Simon Zhang:** Writing – review & editing, Writing – original draft, Software, Methodology, Investigation, Formal analysis, Data curation, Conceptualization. **Cat Shore-Lorenti:** Writing – review & editing, Resources, Project administration, Methodology, Formal analysis, Data curation. **Cherie Chiang:** Writing – review & editing, Validation, Methodology, Investigation. **Frances Milat:** Writing – review & editing, Validation, Methodology, Investigation. **Peter R. Ebeling:** Writing – review & editing, Validation, Supervision, Project administration, Funding acquisition, Conceptualization. **Roger Zebaze:** Writing – review & editing, Writing – original draft, Validation, Supervision, Software, Resources, Project administration, Methodology, Investigation, Funding acquisition, Formal analysis, Data curation, Conceptualization.

## Ethics declaration

Written informed consent to take part in the study and to publish the article has been obtained from all participants or their legal representatives. The privacy rights of participants have been observed.

This study was performed in compliance with relevant laws, regulatory frameworks and guidelines where the research took place.

This study was approved by the Monash Health Human Research Ethics Committee.

(Approval No. HREC/76946/MonH-2021-282773(v2))

This research follows the CARE guidelines and the CARE checklist.

## Declaration of competing interest

R. Zebaze is a co-inventor of ALIGNOGRAM 1.0 and participated in the analysis and unblinded interpretation of its findings, creating potential for bias. Independent external replication and blinded validation are therefore needed. This study was supported by an investigator-initiated research grant from 10.13039/100006396Alexion Pharmaceuticals (ISR 200129). The sponsor had no role in study design, conduct, data analysis, interpretation, or preparation of the manuscript. The remaining authors declare no competing interests.

## Data Availability

The authors do not have permission to share data.
