# Supplementary material for: Automated detection and quantification of bone disorganization in hypophosphatasia before and after asfotase alfa: A case report
Source: Bone Rep. 2026 Oct 23;31:101963. doi: 10.1016/j.bonr.2026.101963 (PMC13722107; doi:10.1016/j.bonr.2026.101963)
Supplement: Supplementary file 1 [file mmc1.pdf]

Hidden or Latent disorganized (misarrangement) lesion

Undetectable without device assistance, even by experts.

Hidden or Latent disorganized (misarranged) lesion

Undetectable without device assistance, even by experts.

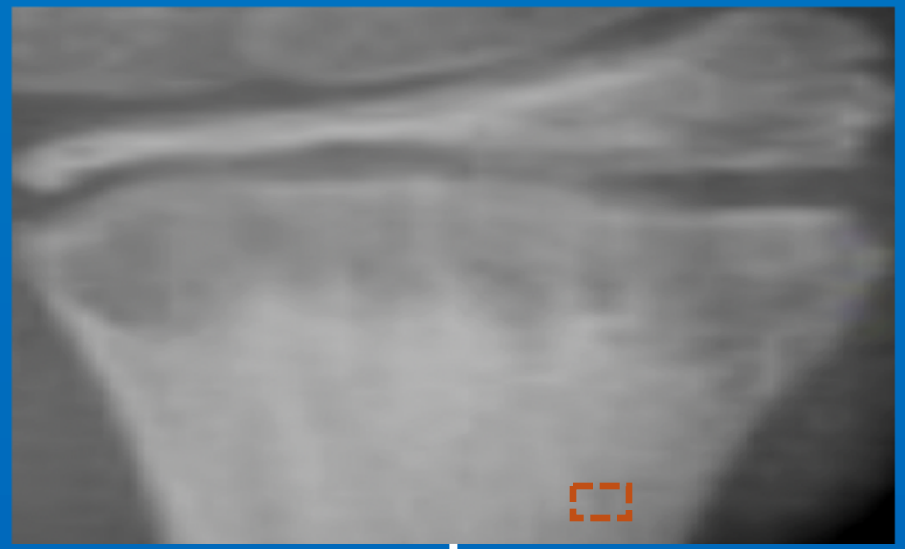

Magnified Distal Radius X-ray Image (16 YO with HPP)

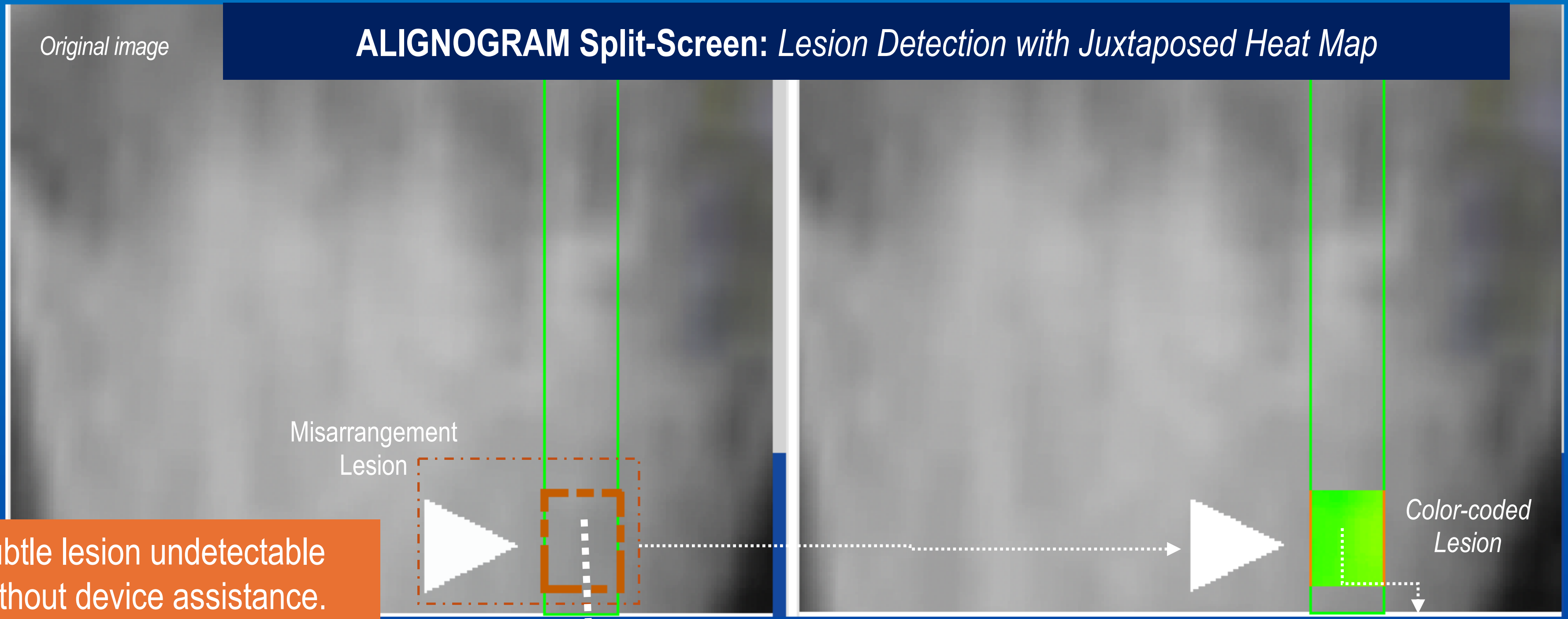

subtle lesion undetectable without device assistance.

ALIGNOGRAM-detected lesion invisible unaided, requires expert processing.

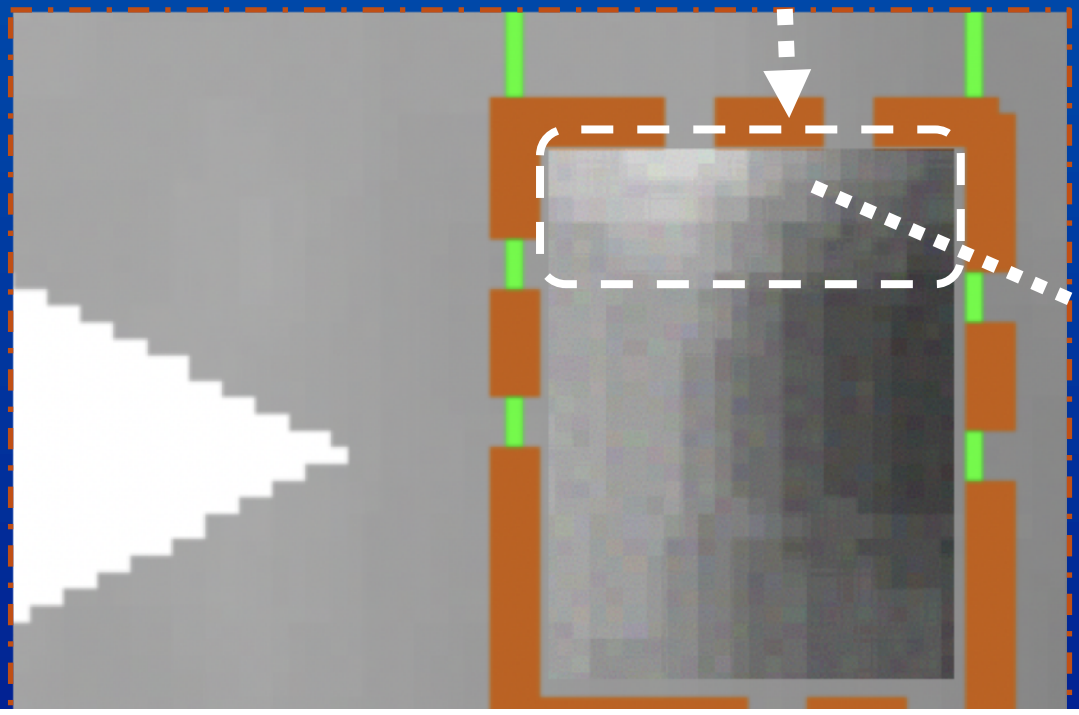

Misarranged opaquer elements (pixels)

Visually undetectable, ALIGNOGRAM Curve-visible.

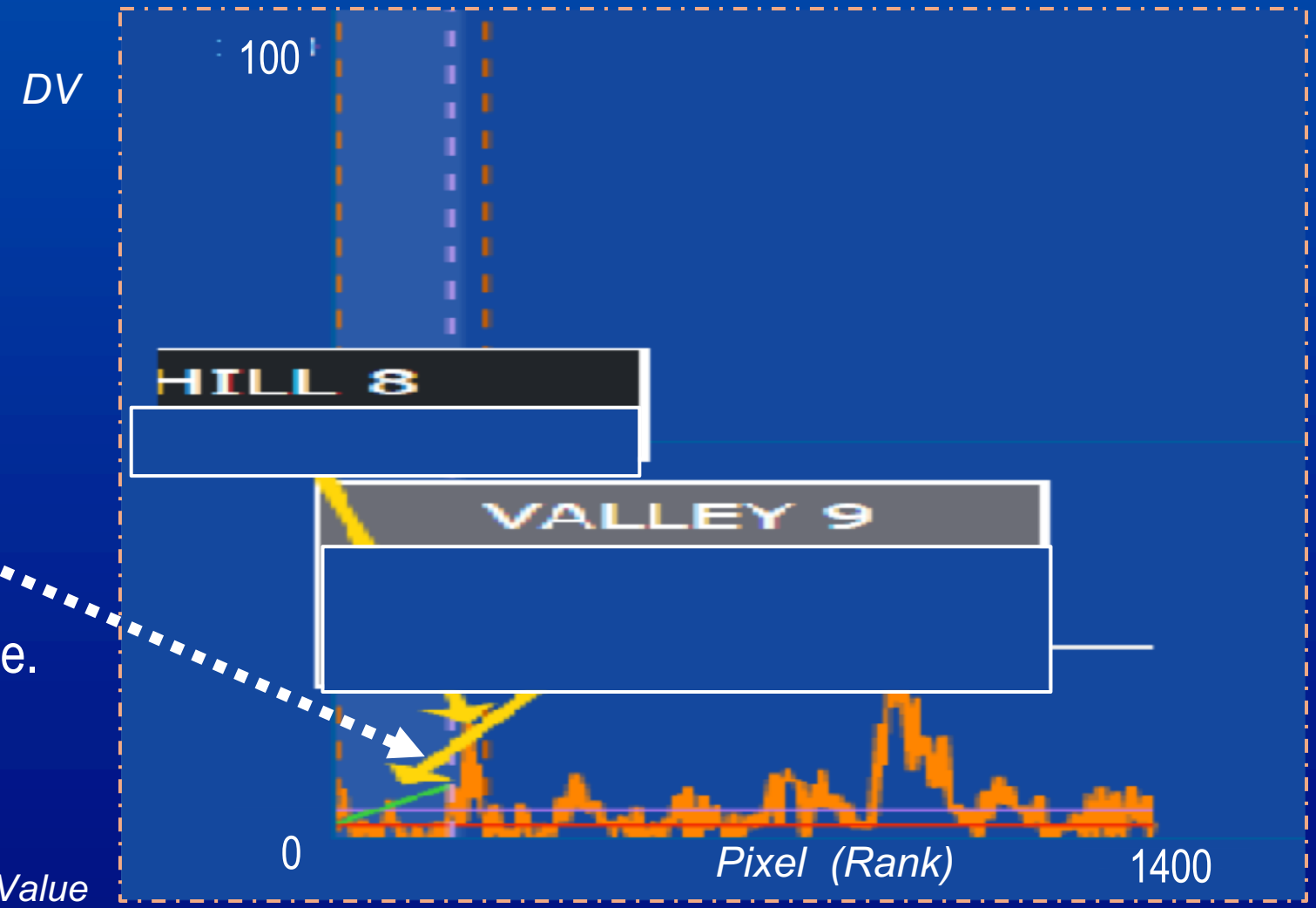

DV= Disorganization Value

‘Distal Radius X-ray Image from Drug Design, Development and Therapy 2018 12 3147-3161‘ Originally published by, adapted, and used with permission from Dove Medical Press Ltd.’

Hidden or Latent disorganized (misarranged) lesion

Undetectable without device assistance, even by experts.

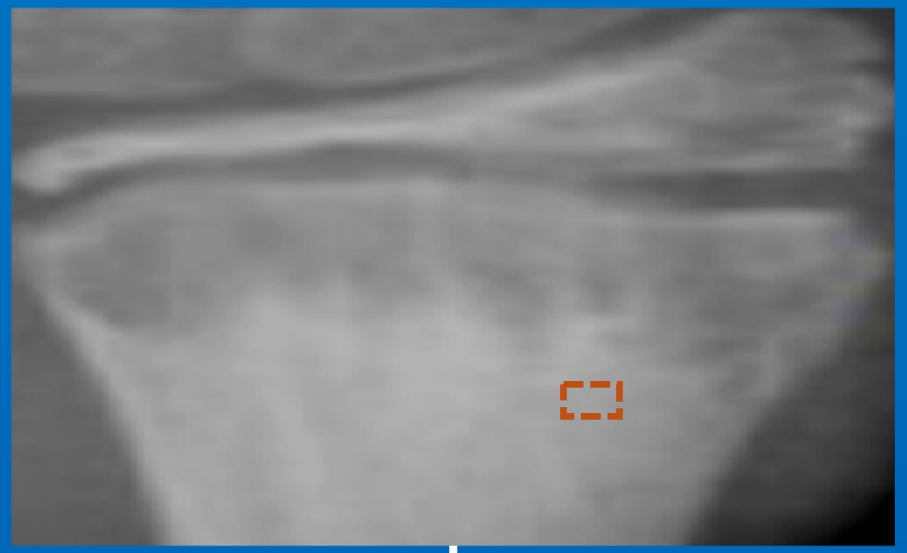

Magnified Distal Radius X-ray Image (16 YO with HPP)

ALIGNOGRAM Split-Screen: *Lesion Detection with Juxtaposed Heat Map*

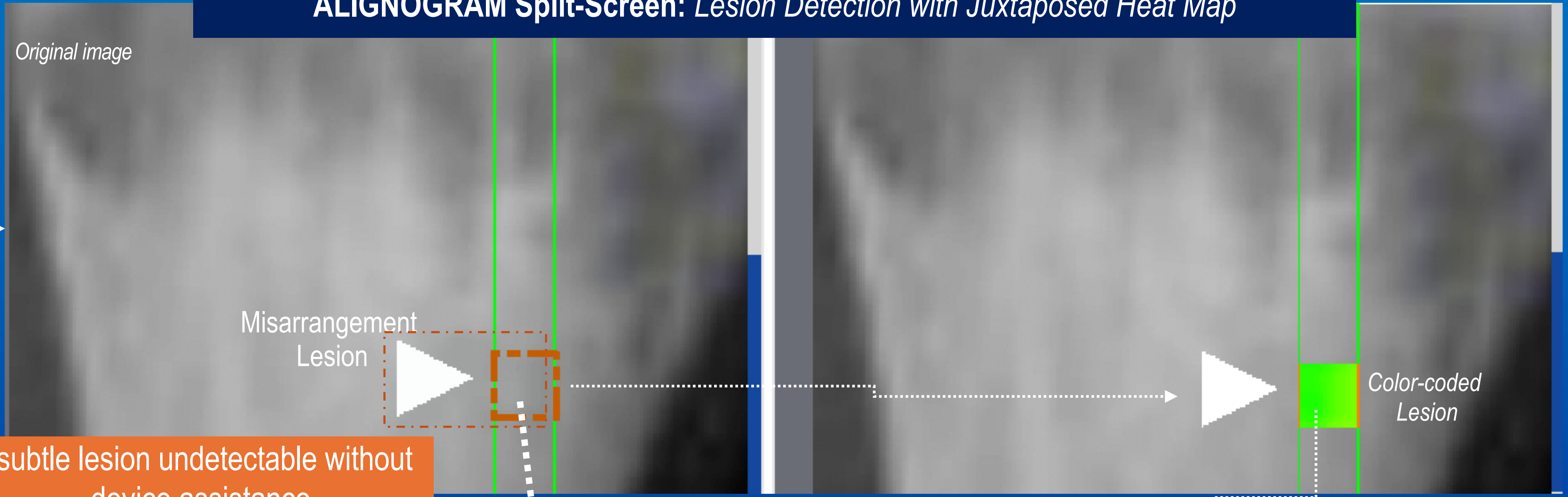

subtle lesion undetectable without device assistance.

ALIGNOGRAM-detected lesion invisible unaided, requires expert processing.

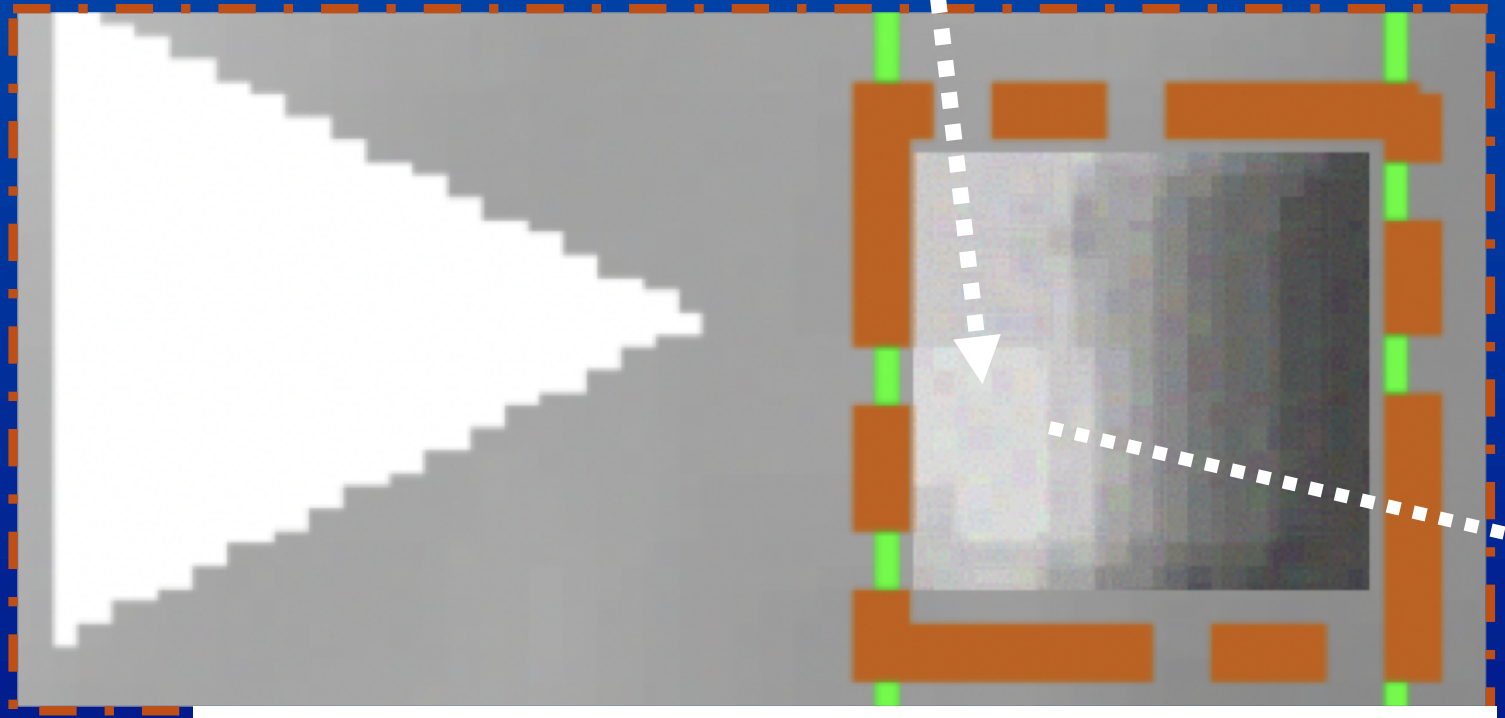

Misarranged lucent & opaque elements (pixels).

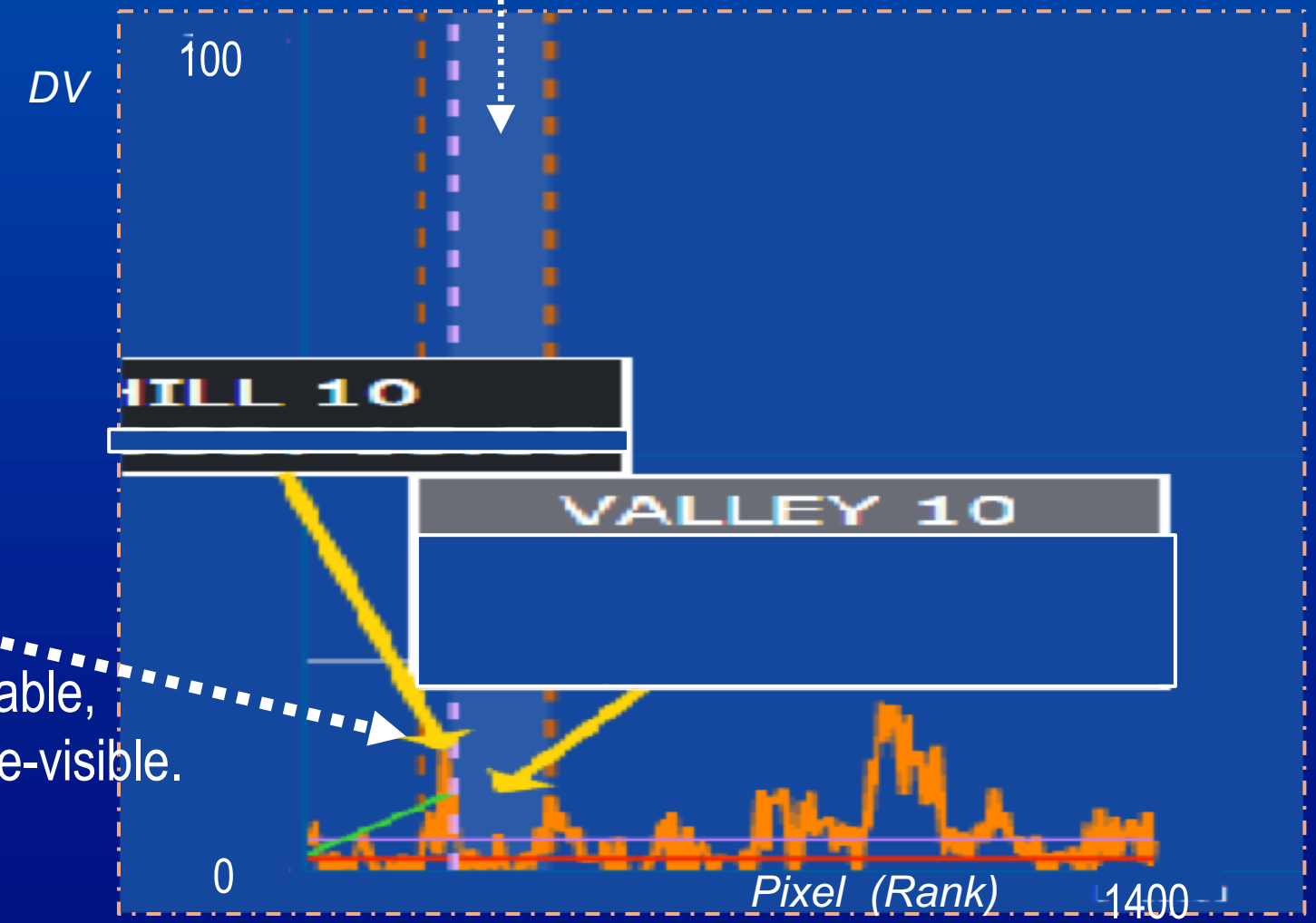

Visually undetectable, ALIGNOGRAM Curve-visible.

'Distal Radius X-ray Image from Drug Design, Development and Therapy 2018 12 3147-3161' Originally published by, adapted, and used with permission from Dove Medical Press Ltd.'

Hidden or Latent disorganized (misarranged) lesion

Undetectable without device assistance, even by experts.

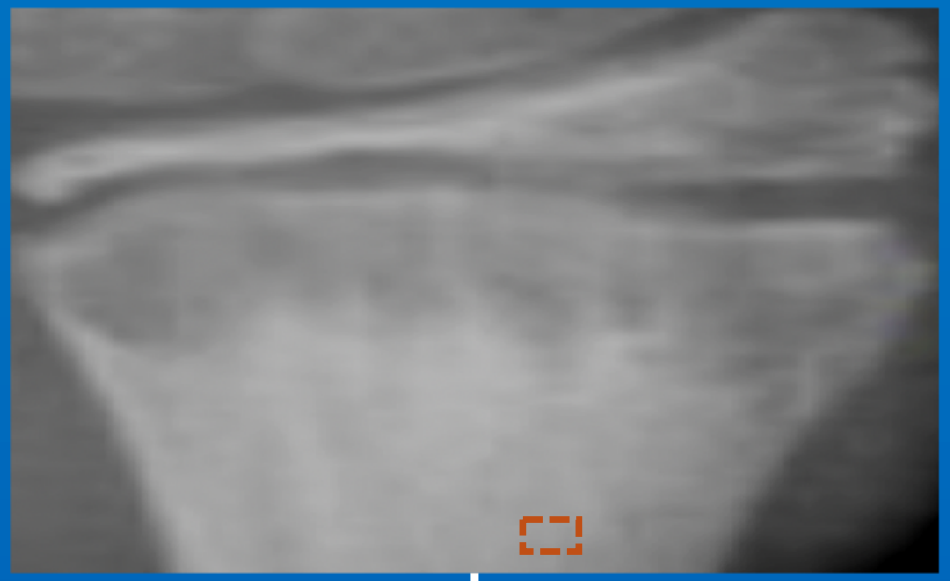

Magnified Distal Radius X-ray Image (16 YO with HPP)

ALIGNOGRAM Split-Screen: *Lesion Detection with Juxtaposed Heat Map*

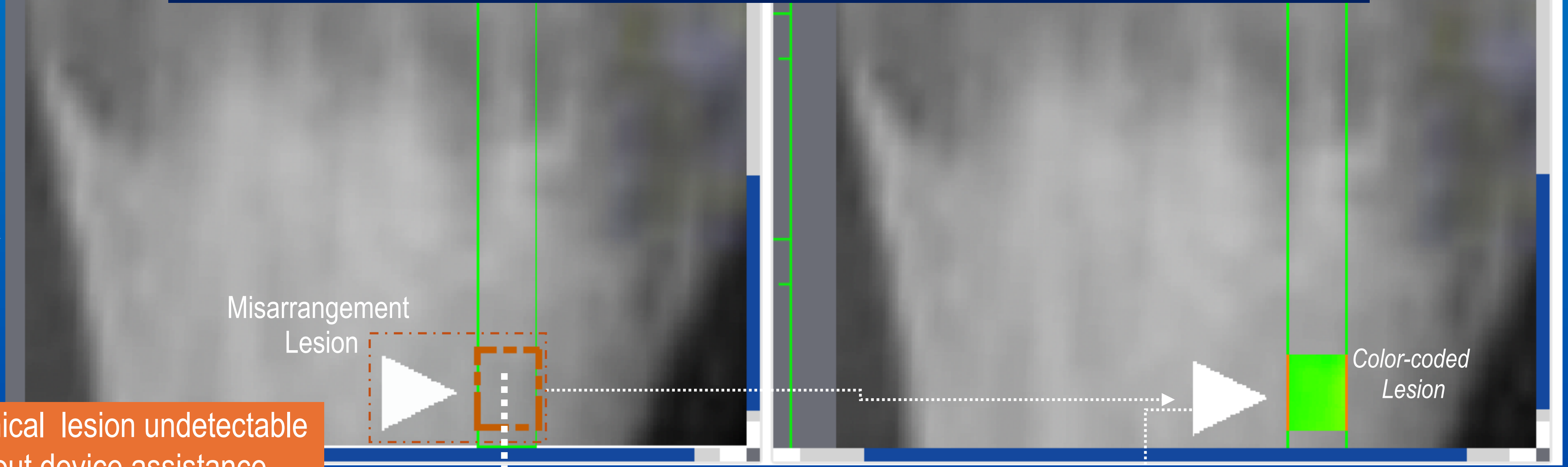

Subclinical lesion undetectable without device assistance.

ALIGNOGRAM-detected lesion invisible unaided, requires expert processing.

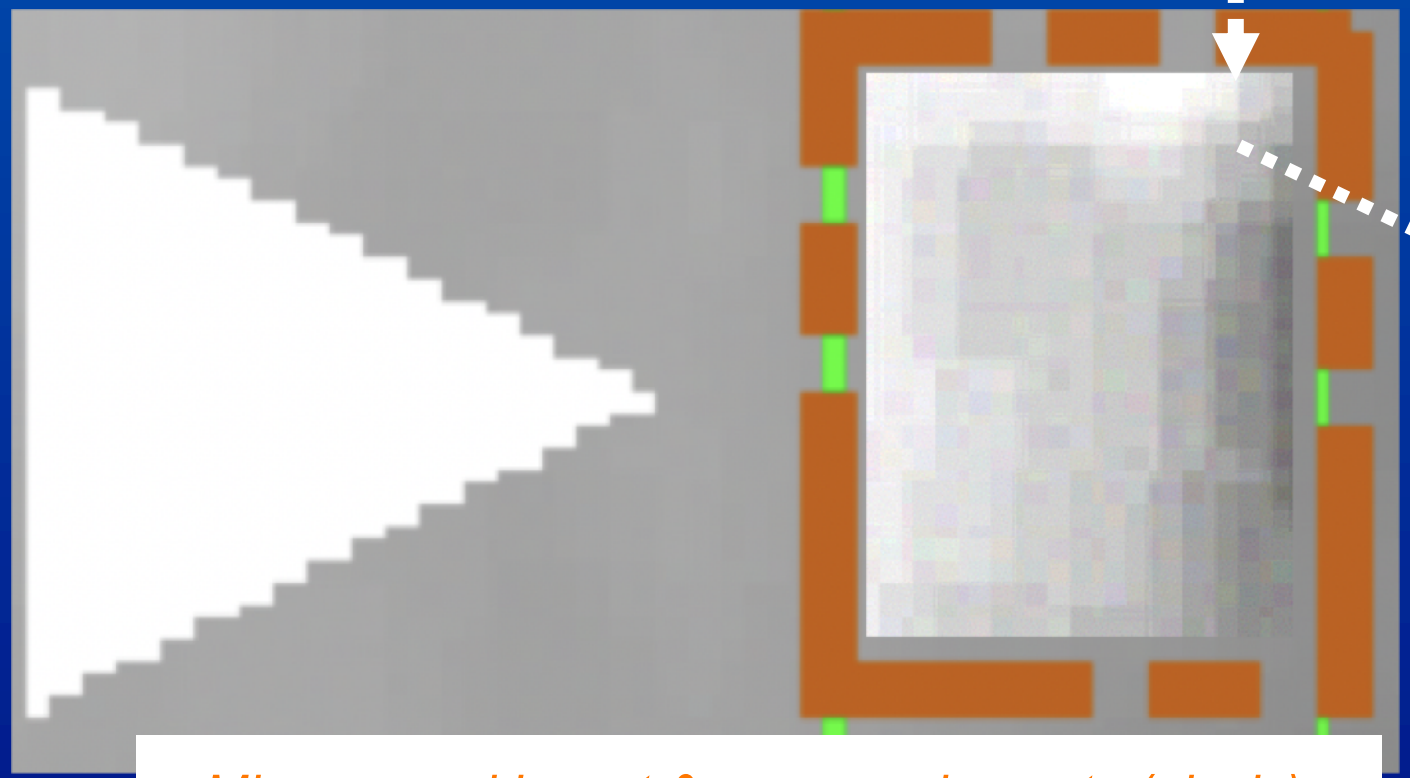

Misarranged lucent & opaque elements (pixels).

Visually undetectable, ALIGNOGRAM Curve-visible.

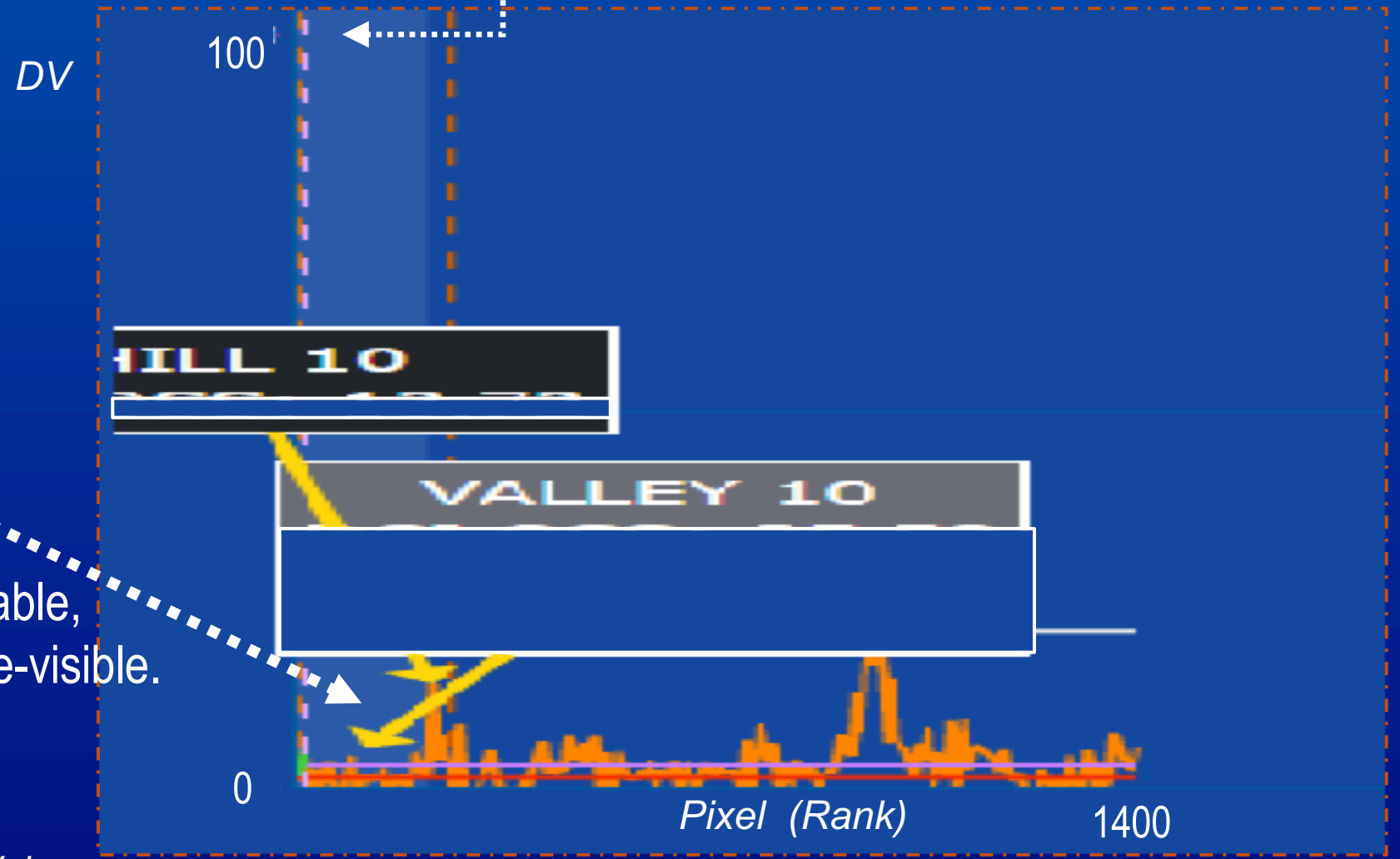

***DV=** Disorganization Value*

Hidden or Latent disorganized (misarranged) lesion

Undetectable without device assistance, even by experts.

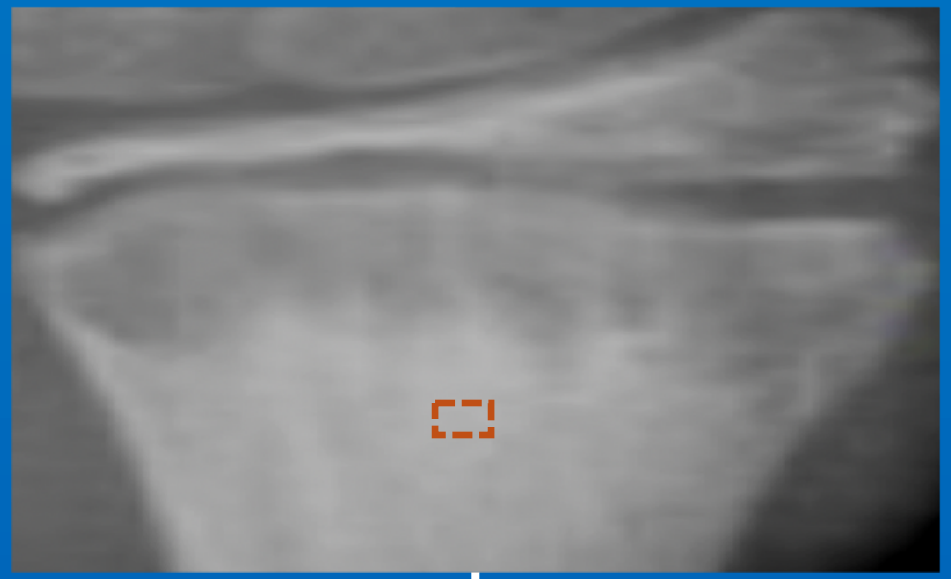

Magnified Distal Radius X-ray Image (16 YO with HPP)

ALIGNOGRAM Split-Screen: *Lesion Detection with Juxtaposed Heat Map*

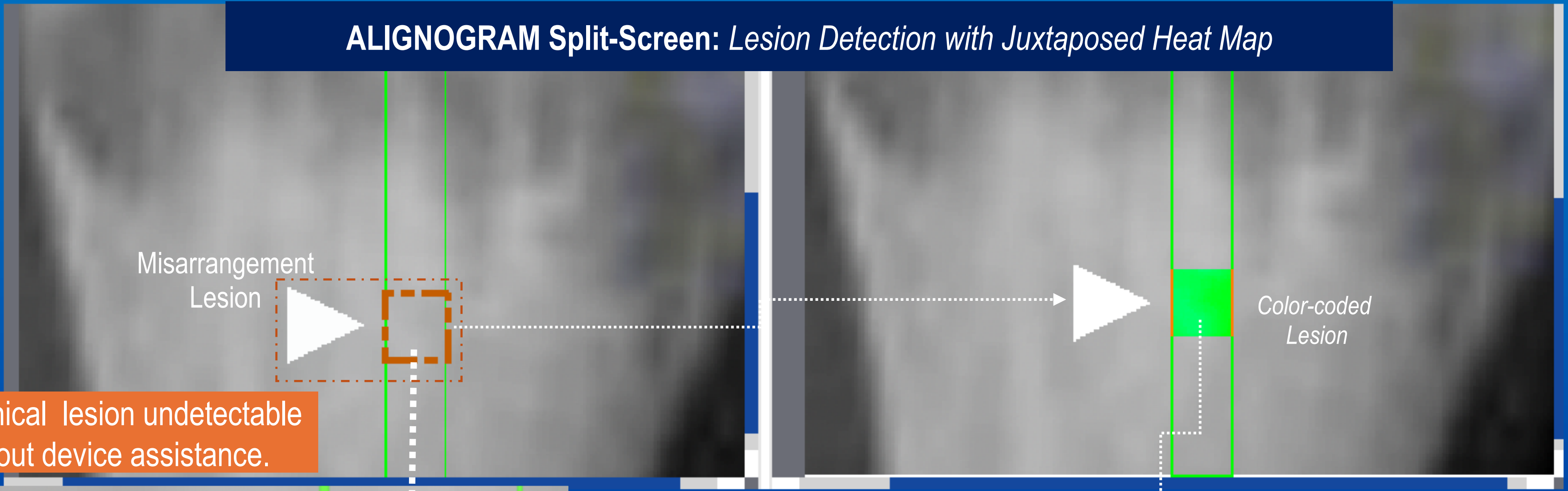

Subclinical lesion undetectable without device assistance.

ALIGNOGRAM-detected lesion invisible unaided, requires expert processing.

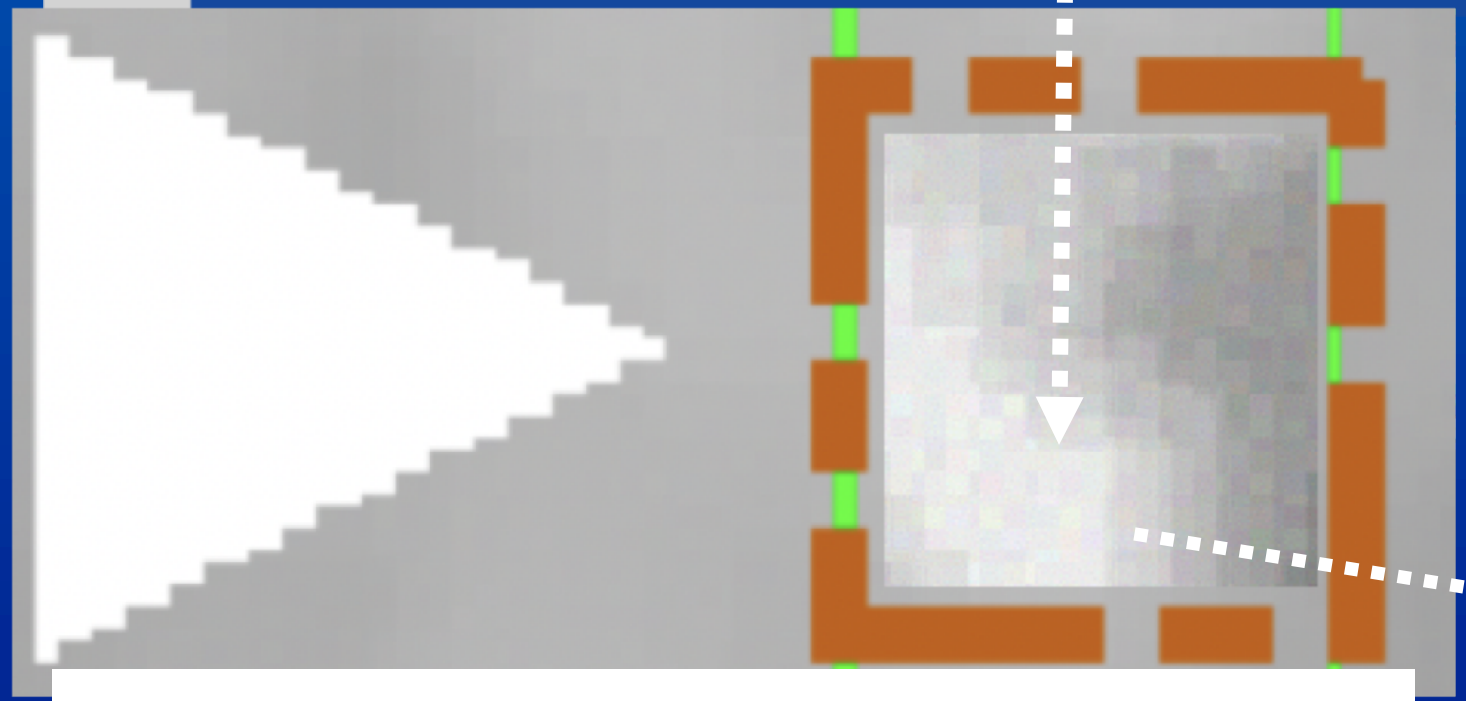

Misarrangement caused by mixture of lucent & opaque elements (pixels).

Visually undetectable, ALIGNOGRAM Curve-visible.

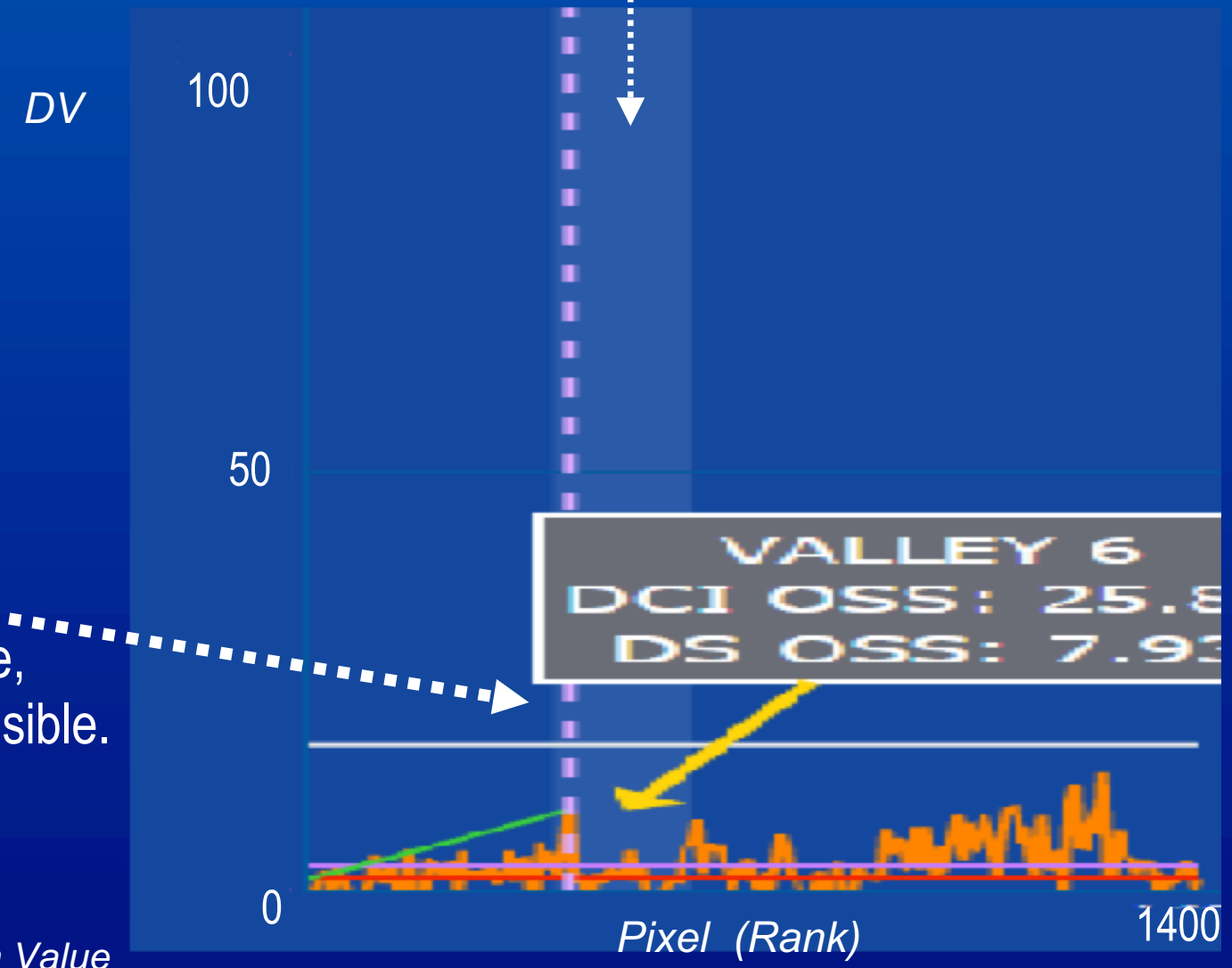

***DV=** Disorganization Value*

'Distal Radius X-ray Image from Drug Design, Development and Therapy 2018 12 3147-3161' Originally published by, adapted, and used with permission from Dove Medical Press Ltd.'

Hidden or Latent disorganized (misarranged) lesion

Undetectable without device assistance, even by experts.

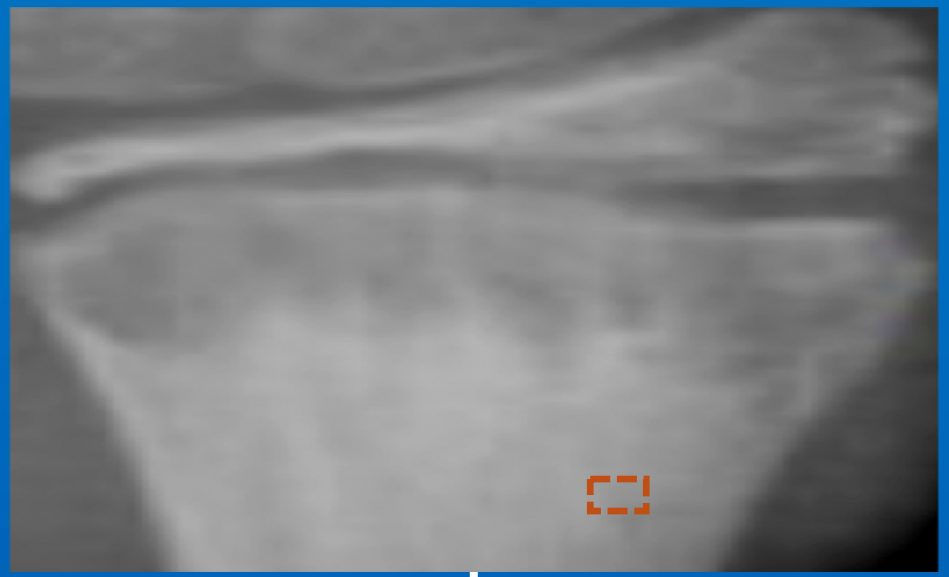

Magnified Distal Radius X-ray Image (16 YO with HPP)

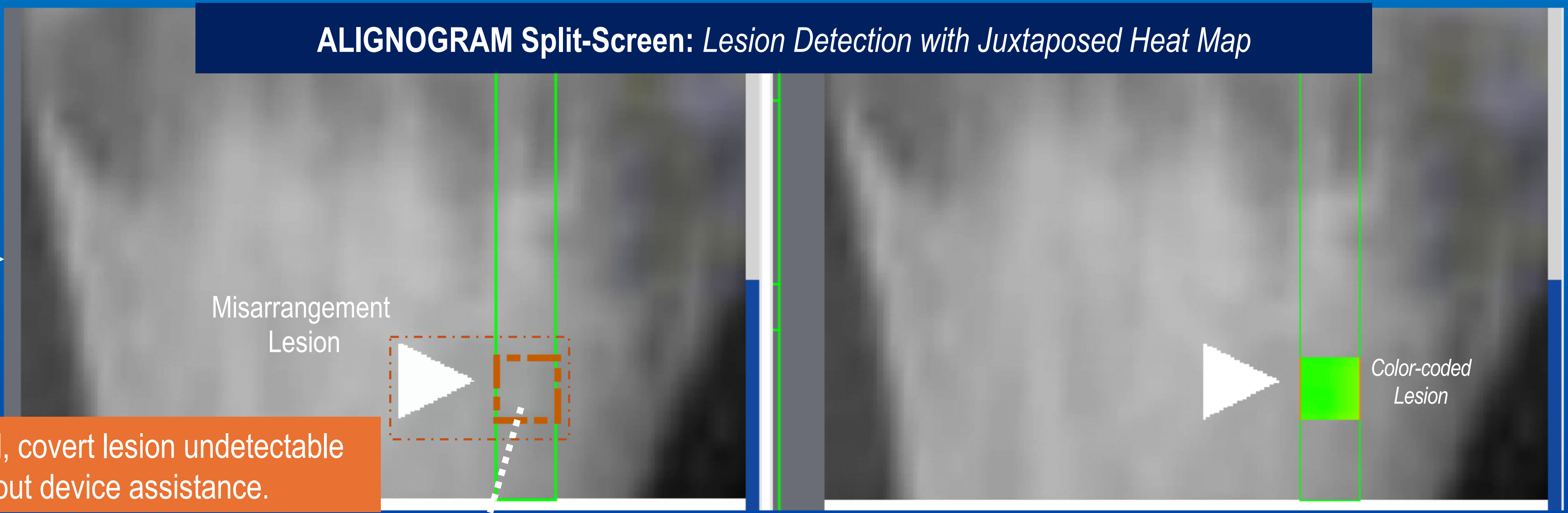

Subclinical, covert lesion undetectable without device assistance.

ALIGNOGRAM-detected lesion invisible unaided, requires expert processing.

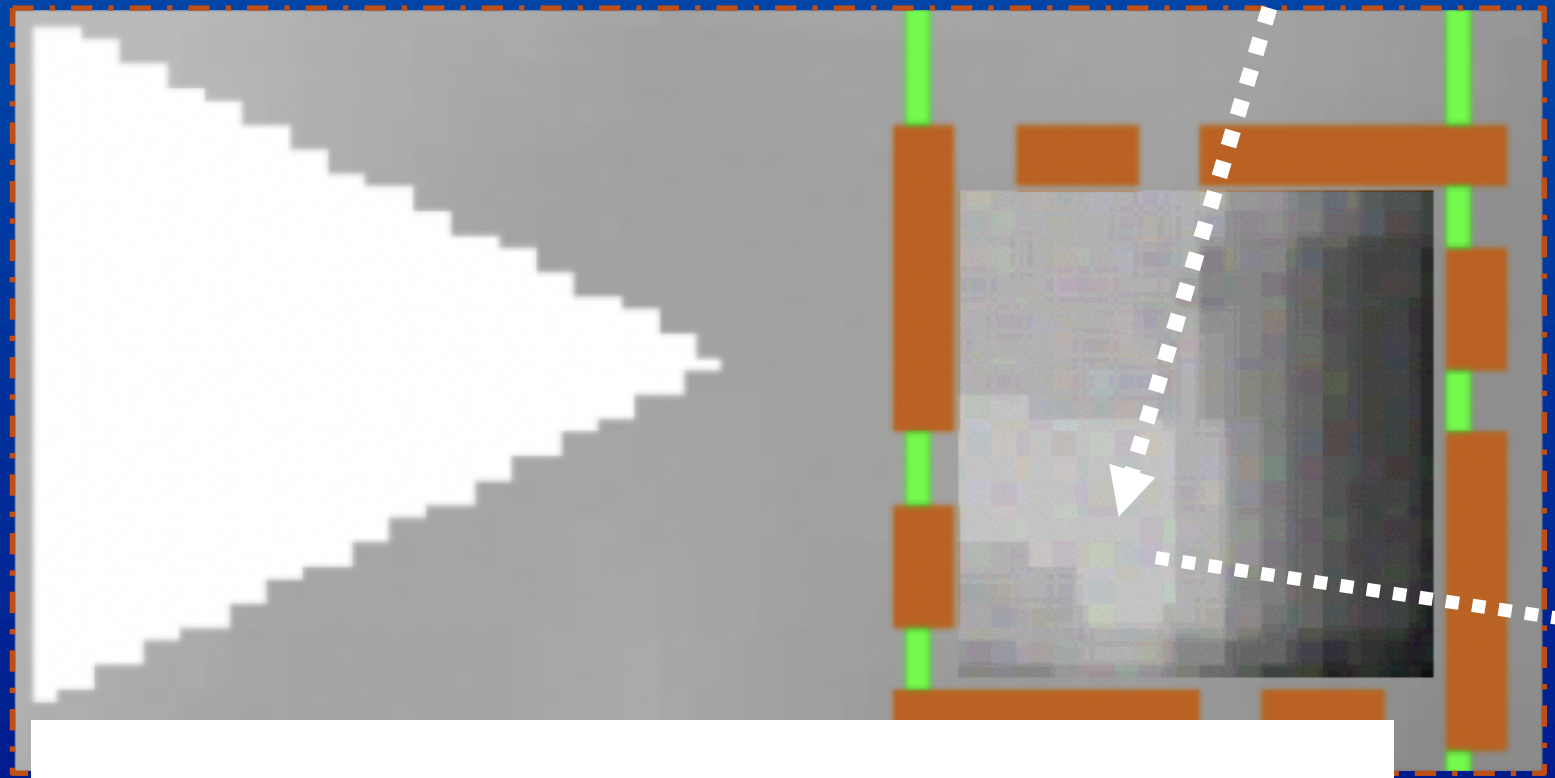

Misarrangement caused by mixture of lucent & opaque elements (pixels).

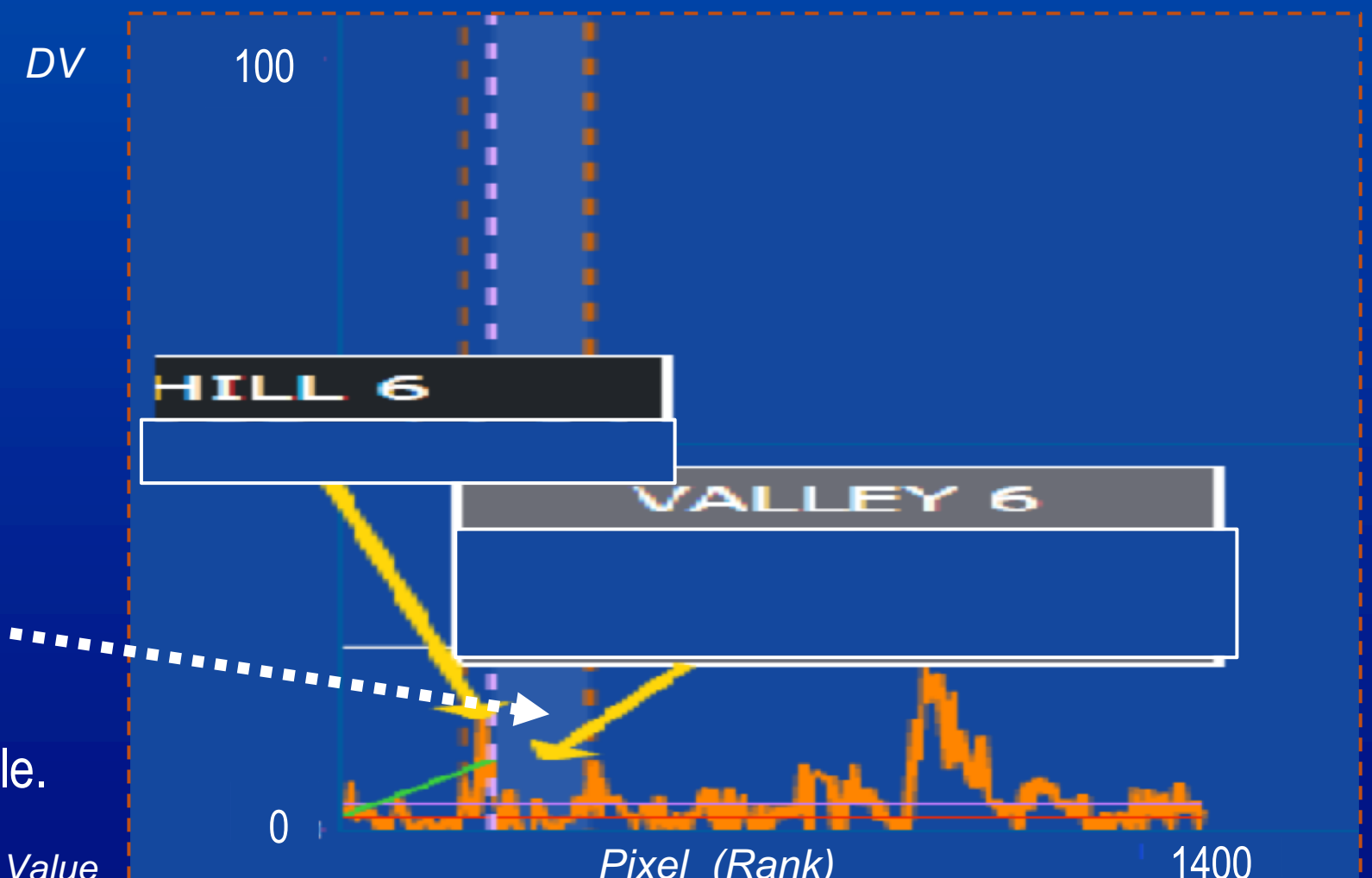

Visually undetectable, ALIGNOGRAM Curve-visible.

DV= Disorganization Value

‘Distal Radius X-ray Image from Drug Design, Development and Therapy 2018 12 3147-3161‘ Originally published by, adapted, and used with permission from Dove Medical Press Ltd.’
